# Clinical significance and gene expression study of human hepatic stellate cells in HBV related-hepatocellular carcinoma

**DOI:** 10.1186/1756-9966-32-22

**Published:** 2013-04-19

**Authors:** Rui Liao, Han Wu, Yong Yi, Jia-Xing Wang, Xiao-Yan Cai, Hong-Wei He, Yun-Feng Cheng, Jian Zhou, Jia Fan, Jian Sun, Shuang-Jian Qiu

**Affiliations:** 1 Liver Cancer Institute, Zhongshan Hospital, Fudan University, Shanghai, China; 2 Key Laboratory of Carcinogenesis and Cancer Invasion, the Chinese Ministry of Education, Shanghai, China; 3 Biomedical Research Center, Zhongshan Hospital, Fudan University, Shanghai, China

**Keywords:** Hepatic stellate cell, Prognosis, Phenotype, Gene expression, Hepatocellular carcinoma

## Abstract

**Background:**

Peritumoral activated hepatic stellate cells (HSCs) are versatile myofibroblast-like cells closely related with hepatocellular carcinoma (HCC) progression. So far, comprehensive comparison of gene expression of human HSCs during hepatocarcinogenesis is scanty. Therefore, we identified the phenotypic and genomic characteristics of peritumoral HSCs to explore the valuable information on the prognosis and therapeutic targets of HBV related HCC.

**Methods:**

A tissue microarray containing 224 HBV related HCC patients was used to evaluate the expression of phenotype markers of HSCs including α-SMA, glial fibrillary acidic protein (GFAP), desmin, vinculin and vimentin. HSCs and cancer associated myofibroblasts (CAMFs) were isolated from normal, peritumoral human livers and cancer tissues, respectively. Flow cytometry and gene microarray analysis were performed to evaluate the phenotypic changes and gene expression in HCC, respectively.

**Results:**

Peritumoral α-SMA positive HSCs showed the prognostic value in time to recurrence (TTR) and overall survival (OS) of HCC patients, especially in early recurrence and AFP-normal HCC patients. Expression of GFAP positive HSCs cell lines LX-2 was significantly decreased after stimulation with tumor conditioned medium. Compared with quiescent HSCs, peritumoral HSCs and intratumoral CAMFs expressed considerable up- and down-regulated genes associated with biological process, cellular component, molecular function and signaling pathways involved in fibrogenesis, inflammation and progress of cancer.

**Conclusions:**

Peritumoral activated HSCs displayed prognostic value in HBV related-HCC, and their genomic characteristics could present rational biomarkers for HCC risk and promising therapeutic targets.

## Background

Cancer is now recognized as a functional organ caused by deregulated oncogenes including proto-oncogenes and tumor-suppress or anti-oncogenes, which may well contribute to certain mutational events in the cancer phenotype [1,2]. Indeed, equally numerous reports have indicated smoldering inflammation in the tumor microenvironment shared the capacity to induce genetic instability or mutation and subsequently fostered the carcinogenic process [3,4]. Therefore, genetic events and phenotypic variation have shed new light on the relationship between inflammation and cancer. Typically, hepatocellular carcinoma (HCC) is a malignancy characterized by a high degree of genetic heterogeneity and often associated with hepatitis viral infection and cirrhosis [5,6]. Of note, some immune/inflammation-related gene signatures were identified as sensitive “readout” screening for high-risk patients likely to experience HCC recurrence after resection [7,8]. Moreover, a subtype of human HCC sharing a gene expression pattern with fetal hepatoblasts has been linked to poor prognosis, which was supposed to be derived from hepatic progenitor cells [9]. This study revealed the malignant biological phenotypes may result from activation of different oncogenic pathways during tumorigenesis and/or different cells of origin including activated inflammatory cells, like tumor-associated macrophages [10], neutrophils [11] and mast cells [12], which may acquire more potent tumor-promoting activities and result in dismal outcome of HCC. Thus, regulation of gene levels in tumor activated inflammatory cells could provide crucial information on the progress and prognosis of HCC.

Interestingly, hepatic stellate cells (HSCs), myofibroblast-like inflammatory cells under activated state, display plastic phenotypes and properties of progenitor cells [13,14]. In a most recent study [15], we found triggering receptor expressed on myeloid cells (TREM)-1, a potential functional gene in HSCs, enhanced the aggressiveness of HCC cells. Moreover, we have previously demonstrated [16] that the density of peritumoral activated HSCs, including their putative functional genes (SPARC, TNC and FAP), were selectively associated with poor prognosis of HCC, revealing that HSCs could reroute the direction from pro-inflammatory response to promoting tumor. Furthermore, a recent integrative genomic analysis revealed that hepatoma cells induced the functional deregulation of relevant gene networks in HSCs, which correlated to the poor outcome of HCC patients [17]. Also, considerable changed gene expression signatures of activated HSCs have been confirmed to have specific contribution to cirrhosis [18-20] and HCC [21]. However, so far, less attention has been paid on the comprehensive comparison of gene expression of human HSCs during hepatocarcinogenesis.

Here, we depicted that peritumoral HSCs were unfavorable predictors in HBV related HCC following resection, especially in early recurrence and AFP-normal HCC patients. To specifically address the possible heterozygous effects and the functional impact of activated HSCs in the aggressive phenotype of HCC, we also characterize the gene expression profile of peritumoral human HSCs and observed numerous regulated genes potentially influencing the malignant behavior of activated HSCs. These alterations presented potential biomarkers and therapeutic targets to interrupt the pivotal pathways in HCC development.

## Material and methods

### Patients and specimens

We randomly selected 224 untreated HCC patients from 2007 who all had hepatitis B history and complete follow-up data until January 2012 (Table 1). Peritumoral hepatic tissues and matched tumor samples from 3 HBV related HCC patients as well as normal tissues from 3 hepatic hemangiomas patients with resection indications and without HBV infection were used for the isolation of HSCs/CAMFs. All samples were anonymously coded in this study complied with the Ethics Review Committee of Zhongshan hospital of Fudan university. Written informed consent was obtained from all clinical patients involved in this study. We excluded patients with acute infection from this study.

**Table 1:** Peritumoral α-SMA expression according to characteristics of 224 hepatitis B virus related HCC patients

**Characteristics**		**Low expression (n = 44) (cell numbers ≤ 72)**	**High expression (n = 180) (cell numbers > 72)**	***p***
Gender	Male	40	152	0.342
	Female	4	28	
Age(years)	≤51	24	94	0.867
	>51	20	86	
ALT(U/L)	≤75	35	162	0.700
	>75	9	18	
AFP(ng/ml)	≤20	18	68	0.731
	>20	26	112	
Cirrhosis	Yes	37	155	0.810
	No	7	25	
Vascular invasion	Yes	8	46	0.446
	No	36	134	
Encapsulation	Yes	24	96	1.000
	No	20	84	
Number	Single	37	155	0.810
	Multiple	7	25	
Size	≤5	38	122	0.015
	>5	6	58	
Differentiation	I-II	41	128	0.002
	III-IV	3	52	
TNM stage	I	37	121	0.028
	II-III	7	59	

α-SMA: α-smooth muscle actin; AFP: alpha fetoprotein; ALT, alanine aminotransferase; TNM, tumor-node-metastasis.

### Tissue microarray design and immunohistochemistry

A tissue microarray (TMA) was constructed and immunohistochemistry was carried out as described previously [15,22]. Under low-power magnification (100X), positive staining cells were screened and photographs of four representative fields were captured under high-power magnification (400X) in Leica DMLA light microscope (Leica Microsystems, Wetzlar, Germany). The positive cell density of each core was counted by two independent investigators blind to clinical outcome and knowledge of the clinicopathologic data. Data were expressed as mean value (±SE) of the triplicate cores taken from each patient. Primary antibodies were mouse anti-human monoclonal antibodies combined with α-SMA (1:100; DAKO), glial fibrillary acidic protein (GFAP 1:100; Cell signaling), desmin (1:50; DAKO), vinculin (1:200; Upstate, Millipore) and vimentin (1:100; Sigma-Aldrich), respectively.

### Collection of tumor conditioned medium (TCM) and generation of tumor-induced activated HSCs in vitro

As described previously [15], tumor conditioned medium (TCM) was collected from HCC cell lines MHCC97L, HCCLM3 and HCCLM6, respectively. Briefly, 5 × 10^6^ tumor cells were seeded into 100-mm dishes containing 10 mL of DMEM with 10% fetal bovine serum for 24 hours and thereafter washed twice with serum-free DMEM, and then cultured in serum-free DMEM. After another 24 hours, the supernatant was centrifuged, filtered and stored at −20°C until use. HSC cell line LX-2 was cultured in T25 flasks (0.6x10^6^) with 5 ml TCM supplemented with 5% FBS for 24 hours.

### Flow cytometric analysis

According to previous report [18,23], four identified phenotypes of activated HSCs including GFAP, fibronectin, CD56 and IL-17R (antibody from ebioscinece, Santa Cruze and R&D Systems, respectively) were used for flow cytometric analysis. Nonspecific IgG of the corresponding class was used as the negative control.

### Isolation and culture of cells

HSCs/myofibroblasts were isolated as our described previously [15]. We isolated HSCs from surgical peritumoral tissues and CAMFs from matched cancer tissues, respectively. Quiescent HSCs were isolated from normal liver tissues from hepatic hemangiomas and prolong culture cells were used as in vitro activated HSCs. HSCs/myofibroblasts were isolated by collagenase-pronase perfusion and subsequent density centrifugation on Nycodenz gradients. After collagenase-pronase digestion, the resulting cell pellets were centrifuged at 50 g for 2 minutes to remove hepatocytes. Before collecting HSCs/CAMFs, obtained cells were seeded for 15 min in serum free medium to allow Kupffer cells attachment. To further purify non-attached cells, magnetic anti-CD45 beads (MACS, Miltenyi Biotec, Germany) were used to deplete contaminating leucocytes. Peritumoral HSCs and intratumoral CAMFs were studied at 24 hours after isolation. HSCs from normal livers were studied at 24 hours after isolation (quiescent HSCs) or after 10 days culture (in vitro activated HSCs) without passage, respectively. CD45 and CD31 positive cells were not found in isolated cells by immunocytochemistry staining, demonstrating no contaminating pan-leucocytes and endothelial cells. HSCs purity was assessed by the autofluorescence property and morphology, the populations were more than 95% pure. Primary cells, HSC cell lines LX-2 (as gifts by professor Jin-sheng Guo in Zhongshan hospital) and three HCC cell lines (MHCC97L, HCCLM3, and HCCLM6) initially established and preserved by our institute [24] were cultured in DMEM supplemented with 10% fetal calf serum (FCS) and 1% penicillin-streptomycin in 95% air and 5% CO_2_ at 37°C.

### Gene expression analysis

Total RNA was extracted from HSCs/CAMFs for microarray analysis. Microarray hybridization was performed using whole human genome oligo array (4 × 44K, Agilent Technologies) based on the manufacturer’s standard protocol. Differentially expressed genes with statistical significance between two groups were identified through volcano plot filtering. The threshold is fold change ≥2.0, p-value <0.05. Pathway analysis and gene ontology (GO) analysis were applied. Finally, hierarchical clustering was performed to show the distinguishable gene expression pattern among samples.

### Quantitative polymerase chain (qPCR) reaction validation of microarray data

A total of 49 genes were confirmed by qPCR as previous protocol [16] using commercially available primer-probe sets (Applied Biosystems, Foster City, CA) and SYBR Green PCR Master Mix (SABiosciences). Primers for these genes are listed in Additional file 1. Expression of GAPDH was used as an internal control. The gene expression was quantified by the 2^-**△△**CT^ method.

### Statistic analysis

Statistical analysis was performed by Student *t* test, Fisher’s exact tests, *χ*^2^ tests, Spearman ρ coefficients tests. The “minimum *p* value” approach [11,12] was used to get an optimal cut-off (high α-SMA expression >72) by X-tile 3.6.1 software (Yale University, New Haven, CT, USA). *P <* 0.05 was considered statistically significant.

## Results

### Expression and predictive value of distinct phenotype markers of HSCs in HCC

**Figure 1:**
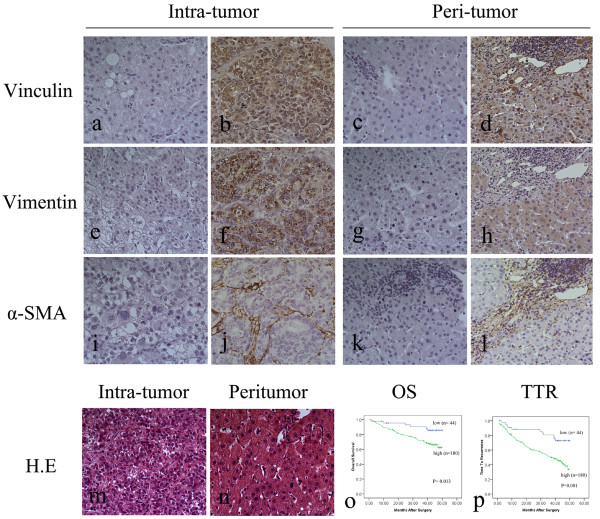
**Images of immunostained cells, HE stain and survival curves for univariate analyses. a**-**l** showed vinculin, vimentin and α-SMA staining cells in intratumoral (**a, b, e, f, i** and **j**) and peritumoral areas (**c, d, g, h, k** and **l**), respectively (x 200). **a**, **c**, **e**, **g**, **i** and **k** were negative controls. **m** and **n** showed HE stain in intratumoral (**m**) and peritumoral areas (**n**), respectively (x 200). High density of peritumoral α-SMA was related to decreased OS (**o**) and TTR (**p**).

Desmin and GFAP were both negatively expressed in all tissue sections. Vinculin and vimentin were expressed ubiquitously on stromal cells and parenchymal cells and no predictive value was found in HCC patients. Consist with previous data [15,16], peritumoral α-SMA was significantly related with poor prognosis of these HBV related HCC patients (cut-off: low ≤ 72, high >72, Figure 1 and Table 2). Moreover, peritumoral α-SMA was associated with tumor size, tumor differentiation and TNM stage. On univariate analysis, vascular invasion, TNM stage as well as peritumoral α-SMA showed prognostic values for both time to recurrence (TTR) and overall survival (OS). Tumor multiplicity was only associated with OS, while AFP and tumor encapsulation can predict TTR, not OS. Then, multivariate analysis was further performed. In addition to peritumoral α-SMA, TNM stage was demonstrated to be related with OS (P = 0.029 and 0.002, respectively) and TTR (P = 0.040 and 0.018, respectively). Significantly, the predictive significance of peritumoral α-SMA was confirmed in early recurrence (≤ 24 months, Table 3) [15] and AFP-normal subgroups (P = 0.014 for OS; P = 0.013 for TTR).

**Table 2:** Prognostic factors for survival and recurrence

**Factor**	**OS**			**TTR**		
	**Univariate**	**Multivariate**		**Univariate**	**Multivariate**	
	***P***	**HR (95% CI)**	***P***	***P***	**HR (95% CI)**	***P***
AFP (≤20 v >20 ng/ml)	NS		NA	0.018		NS
Tumor number (single v multiple)	0.032	2.199(1.209-4.003)	0.010	NS		NA
Vascular invasion(yes v no)	0.008		NS	0.014	1.690(1.011-2.823)	0.045
Tumor encapsulation (yes v no)	NS		NA	0.048		NS
TNM stage (IvII- III)	0.001	2.175(1.326-3.566)	0.002	0.004	1.834(1.111-3.028)	0.018
Peritumoral α-SMA density (low v high)	0.013	2.559(1.101-5.949)	0.029	0.001	2.424(1.040-5.650)	0.040

Univariate analysis: Kaplan-Meier method; multivariate analysis: Cox proportional hazards regression model. Abbreviations: OS: overall survival; TTR: time to recurrence; HR: Hazard Ratio; CI: confidence interval; AFP: alpha fetoprotein; TNM: tumor-node-metastasis; α-SMA: α-smooth muscle actin; NA: not adopted; NS: not significant.

**Table 3:** Prognostic factors for early and late recurrence

**Factor**	**Early recurrence**			**Late recurrence**		
	**Univariate**	**Multivariate**		**Univariate**	**Multivariate**	
	***P***	**HR (95% CI)**	***P***	***P***	**HR (95% CI)**	***P***
AFP(ng/ml)(≤20 v >20)	0.006	1.752(1.035-2.966)	0.037	NS		NA
Tumor size (≤5.0 v >5.0)	<0.001	2.591(1.631-4.116)	<0.001	NS		NA
Vascular invasion(yes v no)	0.011		NS	NS		NA
TNM stage (IvII- III)	0.012		NS	NS		NA
Peritumoral α-SMA density (low v high)	0.002	3.148(1.263-7.844)	0.014	NS		NA

Univariate analysis: Kaplan-Meier method; multivariate analysis: Cox proportional hazards regression model. Abbreviations: HR: Hazard Ratio; CI: confidence interval; AFP: alpha fetoprotein; TNM: tumor-node-metastasis; α-SMA: α-smooth muscle actin; NA: not adopted; NS: not significant.

### Secretion of HCC cells lines partly affected the phenotype modulation of HSCs

**Figure 2:**
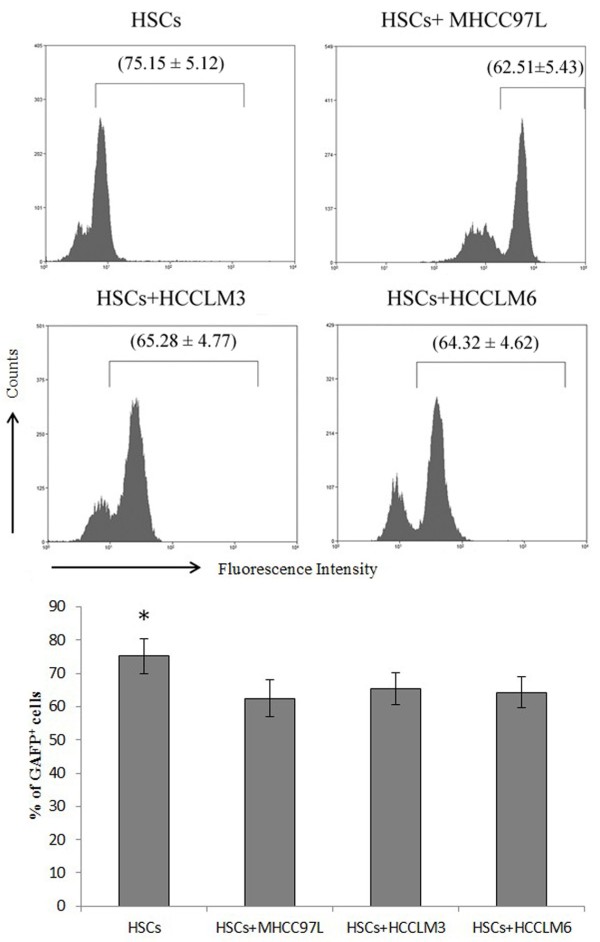
**The frequency of GFAP**^**+ **^**hepatic stellate cells (HSCs) after stimulation with tumor conditioned medium (TCM) from hepatocellular carcinoma (HCC) cell lines MHCC97L, HCCLM3 and HCCLM6 which was determined by flow cytometry.** The relative quantitation was also shown. *P <0.01 compared with HSCs exposed to TCM from HCC cell lines.

Investigated phenotype markers of HSCs showed completely different expression patterns in HCC tissues. Thus, flow cytometric analysis was use to further evaluate the early effects on HSCs (HSC cell line LX-2) response to HCC cells stimulation in vitro. Strikingly, similar to the results of immunohistochemistry, the frequency of GFAP^+^ HSCs was decreased exposed to TCM from HCC cell lines MHCC97L, HCCLM3 and HCCLM6 (Figure 2, P < 0.01). Other investigated biomarkers showed no significance.

### Global comparison in gene expression between different activated/quiescent phenotypes of HSCs and CAMFs

**Figure 3:**
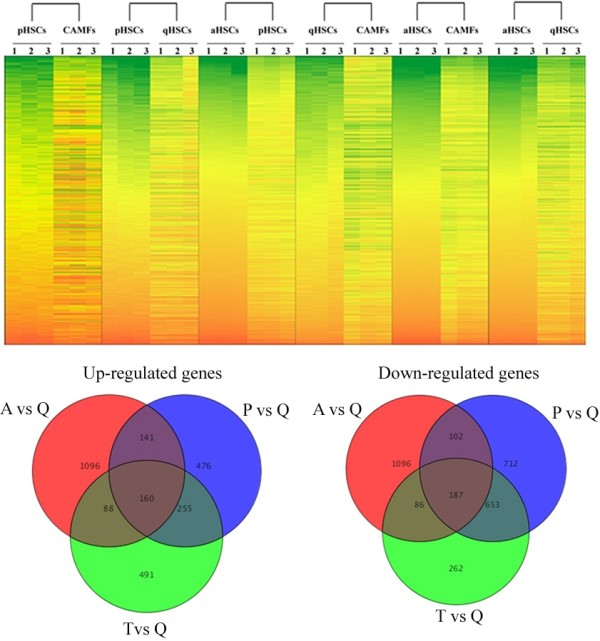
**Gene expression patterns between peritumoral activated hepatic stellate cells (pHSCs), intratumoral cancer associated myofibroblasts (CAMFs), culture-activated HSCs (aHSCs) and quiescence HSCs (qHSCs), respectively.** Each panel of 3 separate cell sample per group (1, 2, and 3) showed hierarchical clustering based on different expression genes represented as a heat map. The Venn plot showed overlapping patterns of probe sets with ≥2-fold up-regulated or down-regulated genes (P < 0.05) in pHSCs (P), CAMFs (T) and aHSCs (A) compared with aHSCs (Q). The number shown in the shared areas represented the common entities.

**Figure 4:**
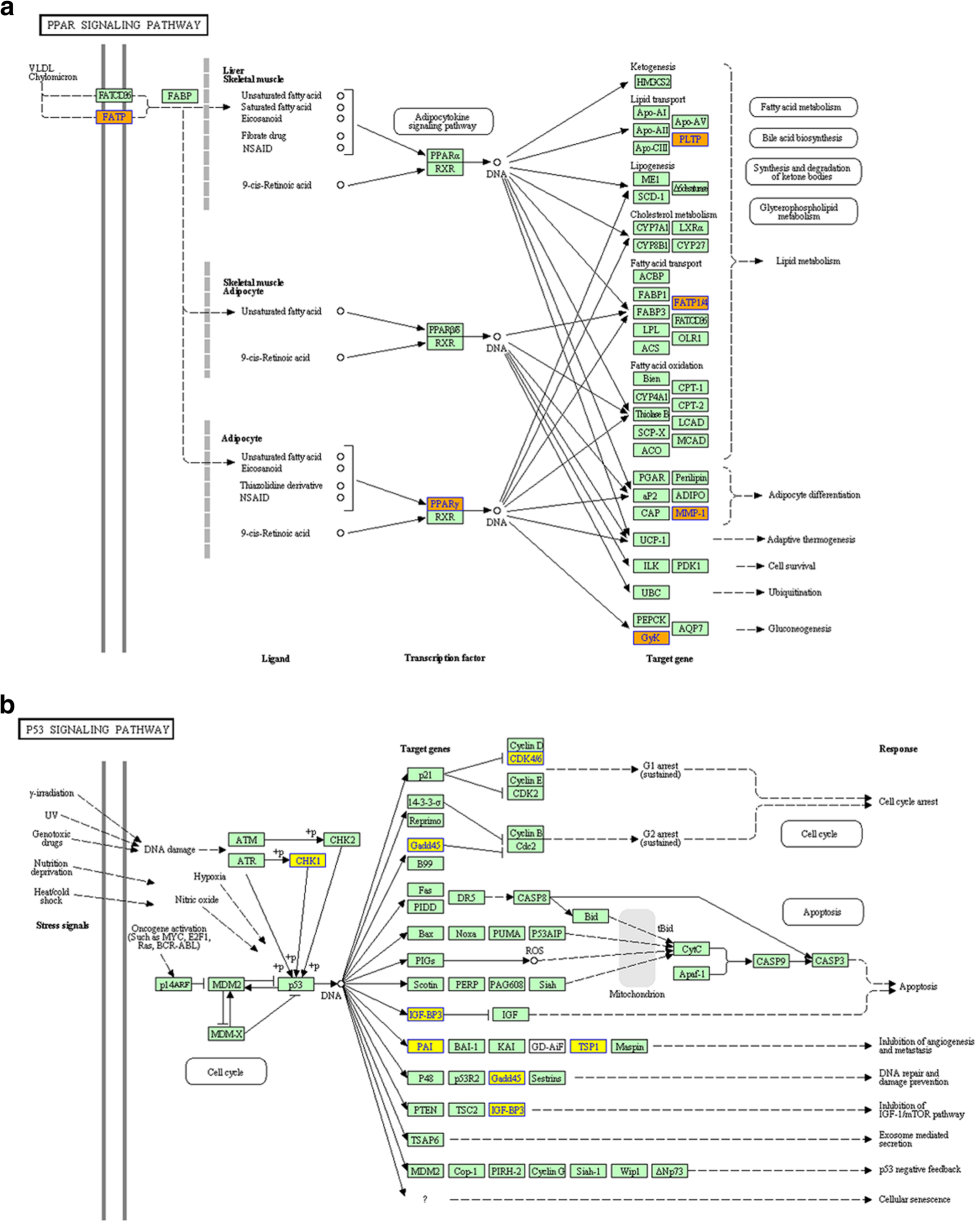
**Pathway analysis showed functional networks identified between peritumoral (P) activated hepatic stellate cells (HSCs) and intratumoral myofibroblasts (T).** Selected significant canonical pathways associated with PPAR signaling (**a**, P vs T upregulation) and P53 (**b**, P vs T downregulation) were shown, respectively. Yellow, orange and green marked nodes represented down-regulated genes, up-regulated genes and no significance, respectively.

Expression levels of 17160 genes were compared between quiescent and activated HSCs and CAMFs from three independent samples per group. Among all significant changed genes (≥2-fold change and p <0.05), there were only 188 upregulated and 467 downregulated genes in peritumoral HSCs compared to intratumoral CAMFs which were from the same HCC patients. Notably, compared with quiescent phenotype HSCs, the same patients-derived culture-activated HSCs yielded as many as 1485 upregulated and 1471 downregulated genes. We found the most significant change happened between peritumoral HSCs/intratumoral CAMFs and culture-activated HSCs (4479 and 3540 upregulated genes, and 3691 and 3380 downregulated genes, respectively) rather than between peritumoral HSCs/intratumoral CAMFs and quiescent phenotype HSCs (1032 and 994 upregulated genes, and 1654 and 1188 downregulated genes, respectively, Figure 3). The levels of correlation between two independent cell populations also displayed these kinds of changes (Additional file 2). Next, we performed a functional analysis associating differentially expressed genes with GO categories, which covered three domains: biological process, cellular component and molecular function. Compared with quiescent HSCs, upregulated genes in peritumoral HSCs and intratumoral CAMFs were investigated to search potential protumor genes (Additional file 3, P < 0.001). In biological process, cell adhesion (e.g. CD209, collagen, type XII, alpha 1), cellular lipid metabolic process (e.g. catalase, platelet-derived growth factor alpha) and other processes related genes were upregulated in peritumoral HSCs and intratumoral CAMFs. Cellular component includes cytoplasmic part (e.g. enolase 2, glucuronic acid epimerase), contractile fiber (e.g. vinculin, matrix metallopeptidase 2), among others. Molecular function consists of protein binding (e.g. vascular endothelial growth factor A, transforming growth factor beta 1), growth factor binding (e.g. oncostatin M receptor, insulin-like growth factor binding protein 6) and so on. Finally, base on the latest KEGG (Kyoto Encyclopedia of Genes and Genomes, http://www.genome.jp/kegg) database, we performed pathway analysis by differentially expressed genes. The p-value (<0.05) denotes the significance of the pathway correlated to the conditions. Lower the p-value, more significant is the pathway. Of note, several well-known pathways in development of HCC such as VEGF [25] (e.g. mitogen-activated protein kinase 13, protein kinase C, beta) and p53 [25] (e.g. cyclin-dependent kinase 6, insulin-like growth factor binding protein 3) signaling pathway related genes were changed significantly in comparison between peritumoral HSCs and CAMFs (Figure 4 and Additional file 4).

### Verification of the DNA microarray results

To validate the results of DNA microarray, some identified genes of interest involved in liver fibrogenesis and hepatocarcinogenesis were assessed by qRT-PCR. Similar up- and down- regulated trends with DNA microarray were detected in the genes encoding key molecules implicated in inflammation (e.g. IL-17RA, TLR-2), tumor invasion and metastasis (e.g. MMP25), adhesion (e.g. CD36, VCAM1), extracellular matrix degradation (e.g. TIMP2), cytoskeletal organization (e.g. ACTG2, ACTA2). Other genes (e.g. COL1A2, MMP1, GK, PPARG) associated with certain pathways such as ECM-receptor interaction and PPAR signaling pathway also confirmed the results obtained in DNA microarray (Additional file 5).

## Discussion

The plasticity of HSCs phenotype and the lack of specific marker proteins hampered an in depth analysis of the nature and functional properties of these fibroblastic cells in human normal and diseased liver. In particular, heterogeneity of phenotypic features among HSCs present in HCC was seldom noticed. In this present study, our immunohistochemical analysis displayed various distribution and expression intensity of four most prominent HSCs phenotype/gene markers including α-SMA, desmin, GFAP and vimentin [14] as well as a recently reported marker vinculin [26], which probably exhibited their different in vivo biological behaviors and cellular response to injurious stimuli in the progress of HCC. Although desmin and GFAP were markers of rat/mouse HSCs [14,27] and GFAP has also been identified as an early marker of human HSCs activation [28,29], our study showed they were not expressed in human HCC tissues. Also, vimentin and vinculin were not specific markers for human HSCs, at least in HCC. These results suggested the complexity and the difference of HCC milieu compared to other chronic liver diseases. Excitedly, as a canonical marker of activated HSCs, high expression of α-SMA still showed specificity in HSCs and a good prognostic performance in HBV related HCC patients, which therefore could provide us a reliable monitoring indicator in at-risk HBV related HCC patients.

In line with our previous studies [15,16], peritumoral HSCs were demonstrated as independent predictors for HCC patients with higher recurrence rate and reduced survival times, also closely related to adverse HCC characteristics like tumor size, tumor differentiation and TNM stage. These data supported the protumor function of activated HSCs. A more important finding was observed that peritumoral HSCs served as unfavorable prognostic predictors in several subgroups including early recurrence group (≤ 24 months) [15] and AFP-normal patients in HBV related HCC. These results implied activated HSCs could participate in intrahepatic metastases probably involved in the conversion of pro-inflammatory response into promoting tumor [15]. Furthermore, for the AFP-normal HCC patients who were difficult to be supervised, peritumoral HSCs were potential monitoring indicators because of their better prognostic values in the AFP-normal group.

In HCC tissues, different expression patterns of phenotype markers of HSCs probably were cellular response to long-term injurious stimuli in HCC microenvironment. Thus, the early effects of HCC on HSCs (HSC cell line LX-2) were further evaluated by flow cytometry. Here, GFAP showed decreased expression in LX-2 after tumor stimulation, which can partly interpret its transform from an activated marker in chronic liver disease [28,29] to negative expression in HCC tissues. Moreover, GFAP was identified as a tumor suppressor gene in astrocytoma [30] and glioma pathogenesis [31]. Loss of GFAP expression in tumor progression could be secondary loss of a differentiation marker and represented a step in tumor development [32].

On base of the clinical analysis results as aforementioned, we conjectured that there could be more potential key molecules or genes in HSCs which were related with their functional properties and triggered their activation during the development of HCC. Although our colleagues [21] recently assessed the features of rat HSCs cultured in conditioned medium of HCC cell lines, no study has directly investigated gene expression patterns of HSCs in liver specimens from patients with HCC. A number of genomic analysis of HSCs have been performed, but majority of these studies were restricted to cirrhosis or chronic liver diseases induced HSC activation [18-20]. Therefore, we investigated gene expression of primary HSCs/CAMFs from normal, peritumoral and intratumoral livers. Detailed genomic analysis will contribute to study of their different roles during hepatocarcinogenesis.

To our knowledge, this is the first study about gene expression profile of HSCs freshly isolated from human HCC tissues. COL1A2, ACTG2 and ACTA2, as typical HSC or myofibroblast-like cell activation markers, were increased significantly in activated HSCs and CAMFs compared to quiescent HSCs. These findings, as well as the validated genes suggested the reliability of DNA microarrays data. Moreover, high correlation coefficients between the same types of cells demonstrated small gene expression variances in each group (Additional file 2: Table S2). Consistent with previous studies [18,20], lower correlation coefficients between culture-activated HSCs and in vivo activated HSCs/CAMF suggested culture-activated HSCs can only partly reflect the underlying gene expression changes of in vivo activated HSCs. Compared with in vivo activated HSCs/CAMFs, different gene expression patterns were detected in culture-activated HSCs probably due to different in vivo stimulus effects and the lack of cell-cell contact and cell–matrix interaction [18]. Importantly, our study identified a large number of previously known and unknown functional genes in activated HSCs/CAMFs during the process of hepatocarcinogenesis. First, peritumoral HSCs and intratumoral CAMFs shared similar gene expression profile (r = 0.936, P < 0.001) and relatively minor gene changes in HCC, which therefore suggested the important roles of these changed genes in hepatocarcinogenesis and the possible evolution from HSCs into myofibroblasts. Compared with upregulated genes, more downregulated genes (188 v 467) in intratumoral CAMFs than peritumoral HSCs may be associated with loss-of-function mutation of genes in intratumoral immunosuppression microenvironments. Second, according to biological process in GO analysis, considerable inflammation/immune response related genes (e.g. IL-18, TNFRSF1B) upregulated in peritumoral HSCs compared with intratumoral CAMFs, indicating activated HSCs were sensitive target cells respond to peritumoral inflammatory stimulus and can be driven into malignant phenotypes fostering the carcinogenic process. Third, pathway analysis of differentially expressed genes further extended the information on the roles of peritumoral HSCs and intratumoral CAMFs in development of HCC. For example, compared with quiescent HSCs, down-regulate of apoptosis related genes in CAMFs may be implicated in their increased proliferative abilities. Compared to CAMFs, lower expression levels of genes in p53 pathway in peritumoral HSCs may attribute to the protumor power of activated HSCs. Fourth, identification of novel genes associated with tumor activated HSCs can benefit an in depth analysis of the nature and functional properties of HSCs in HCC. However, further studies need to test these hypotheses.

Recent epidemiologic data indicate that one of the most important risk factors for HCC development is HBV infection, especially in east Asian [33,34]. Here, in absence of a direct association between HBV infection and HSCs activation, but we highlighted HSCs function as regulators in inflammation-mediated liver injury after HBV infection. An in-depth comparison with other etiologies including hepatitis C virus or alcohol-related HCC could find the association between HBV and HSCs activation. Consist with previous survey [34], our most tissue samples were obtained from patients with typical cirrhosis (192/224, Table 1). Accordingly, we conjecture that cirrhosis might influence the gene expression level in HSCs to a great extent. Further investigation in HCC patients with different grades of fibrosis may provide further insight into the mechanisms of malignant transformation from fibrosis and cirrhosis to HCC.

## Conclusions

In conclusion, we demonstrated that peritumoral activated human HSCs were poor prognostic factors for HBV related HCC after resection, especially in early recurrence and AFP-normal subgroups. Moreover, we showed for the first time that in HCC milieu, peritumoral HSCs markedly expressed fibrogenesis and hepatocarcinogenesis related genes. In this regards, these alterations had potential to be responsible for the acquirement of malignant phenotypes and behavior of activated HSCs during the process of HCC, therefore providing us available multi-target to constitute a promising therapeutic strategy for HCC.

## Competing interests

The authors declare that they have no competing interests.

## Authors’ contributions

RL and HW conceived and designed the experiments. YY, JXW and HWH contributed to the acquisition of the data, XYC has made substantial contribution to collected tissue samples, JZ, YFC, JF, participated in study design and coordination, RL, JS and SJQ contributed to data analysis and interpretation and drafted the manuscript. All authors have read and approved the final manuscript.

## Supplementary Material

Additional file 1: Table S1 Primers for qRT-PCR. Click here for file

Additional file 2: Table S2 Spearman rank correlation coefficient on all targets value. Click here for file

Additional file 3: Table S3 Representative genes in Gene Ontology analysis in different cell phenotypes. Click here for file

Additional file 4: Table S4 Representative genes in pathway analysis in different cell phenotypes. Click here for file

Additional file 5: Table S5 qRT–PCR validated genes in Gene Ontology analysis and pathway analysis in different phenotype cells. Click here for file
