# Supplementary material for: Clinical significance and gene expression study of human hepatic stellate cells in HBV related-hepatocellular carcinoma
Source: J Exp Clin Cancer Res. 2013 Apr 19;32(1):22. doi: 10.1186/1756-9966-32-22 (PMC3654985; doi:10.1186/1756-9966-32-22)
Supplement: Additional file 1: Table S1 — Primers for qRT-PCR. [file 1756-9966-32-22-S1.docx]

**Table S1 Primers for qRT-PCR**

| GENE |  | 5'-3'Primer sequence | PCR product size (bp) |
| --- | --- | --- | --- |
| IL17RA | F | GGTGGTGGGTCAGTGAAGAT | 236 |
|  | R | AGGCATAGGAATGAAGGGC |  |
| COL1A2 | F | ACTACCTCAAAACACTTTCCCAT | 200 |
|  | R | ACACCACACGATACAACTCAATAC |  |
| ACTG2 | F | CAAAGACACACCAGCCCTCA | 167 |
|  | R | GCATCATCTCCTGCGAAGC |  |
| ACTA2 | F | CCCTTGAGAAGAGTTACGAGTT | 145 |
|  | R | ATGATGCTGTTGTAGGTGGTT |  |
| CD81 | F | ATGATGACGCCAACAACGC | 127 |
|  | R | CTGCCCGAGGGACACAAAT |  |
| MMP25 | F | TGAGGACAGAAGGCAGGAGACT | 90 |
|  | R | ATGGAAAAGAAACTGACAGAGGC |  |
| SGK1 | F | AAGGGCAGTTTTGGAAAGGTT | 189 |
|  | R | AGTCTGGAAAGAGAAGTGAAGGC |  |
| MMD | F | GCTTGCCTTTAGATCGGGT | 259 |
|  | R | GGAGTGGTTTGGTTGGTGTT |  |
| GPX3 | F | GCCTACAGGTATGCGTGATT | 192 |
|  | R | CTGGTTTTTCCTTTGGGTTT |  |
| TIMP2 | F | GCCGCTCAAATACCTTCACA | 222 |
|  | R | TTACGGCAGCAAGTCCAATA |  |
| CD83 | F | GCTGGCTCTGGTTATTTTCTACT | 265 |
|  | R | AGCGTAGGCTCATTCTTCTCTC |  |
| F3 | F | TCCCCAGAGTTCACACCTTAC | 163 |
|  | R | AGTCCTTGCCAAAAACATCC |  |
| MMP10 | F | ACGTTGGTCACTTCAGCTCCT | 147 |
|  | R | TCACCTCTTCCCAGACTTTCAG |  |
| PTPN2 | F | CATTTCTGGCTTATGGTTTGG | 119 |
|  | R | AGCATCTCTTGGTCATCTGTTG |  |
| SOCS3 | F | GTATTCTGTGTCAGGTATTGGGC | 225 |
|  | R | GACTTGGATTGGGATTTTGTTG |  |
| IGFN1 | F | AGGAAAATGGGAGTGAGAAGAT | 180 |
|  | R | CTGAAGCGAAGAAGAAAGAAAC |  |
| CCL20 | F | CAGACCGTATTCTTCATCCTA | 145 |
|  | R | ATATATTTCACCCAAGTCTGTTT |  |
| CXCL2 | F | CCCAAACCGAAGTCATAGCC | 154 |
|  | R | CAGGAACAGCCACCAATAAGC |  |
| TLR2 | F | GGTCCTGTGCCACCGTTTC | 84 |
|  | R | GCTTTCCTGGGCTTCCTTTT |  |
| TNF | F | TGCCCCAATCCCTTTATTACC | 124 |
|  | R | CGAAGTGGTGGTCTTGTTGC |  |
| HMGB4 | F | CAAGTAACCAACCAATACGAGC | 110 |
|  | R | GGCATTGTTGAGTCAGAGGAG |  |
| BCL2L2 | F | GCCGCCTTGTAGCCTTCT | 103 |
|  | R | CGTCCTCACCTACCACCG |  |
| VEGFC | F | GCTTCTTCTCTGTGGCGTGTT | 298 |
|  | R | TCTGTCCTTGAGTTGAGGTTGG |  |
| COL5A1 | F | CTCCCTGCTTTCTTTATCCTGT | 107 |
|  | R | AGTGTGCTTGGCTATCCTGC |  |
| TGFB1I1 | F | AACCAGCCCATCCGACAC | 78 |
|  | R | GCAACTGACGCAGCAGAAAT |  |
| IL17RE | F | CCTTGGTGTGTGCGAGTCT | 224 |
|  | R | GAGGGGATGGAAATGTGATG |  |
| IL6 | F | TGAGGAGACTTGCCTGGTGAA | 336 |
|  | R | AATCTGAGGTGCCCATGCTAC |  |
| TLR5 | F | TCATGGTGGTGGTTGGGTC | 251 |
|  | R | TTGTGGCTTGAGATAAGTTGGA |  |
| CXCL6 | F | TGAGTTTCCTGCCAGTCGG | 70 |
|  | R | ACTTTGGTTTCCTCGTGCC |  |
| PDGFA | F | AAGAACTATGCGTCAACCAATC | 74 |
|  | R | TGTCACTCAGCCACAAACG |  |
| MMP3 | F | AAGGGAACTTGAGCGTGAA | 78 |
|  | R | TAAGCAGCAGCCCATTTG |  |
| ACTR2 | F | CCCGACAAGAGTACCAAGAAAAG | 178 |
|  | R | AGTCAAAGGGCAGAGAGAGGC |  |
| AKT3 | F | TGAAAACAGAACGACCAAAGC | 168 |
|  | R | TCATTCTCTCCTCTTCTTGCCT |  |
| BMP2K | F | ACTCTGCTACTACTGCCACTCC | 219 |
|  | R | GCTGTAATCTCCAATCTCCCT |  |
| CD36 | F | TGAGAGAACTGTTATGGGGCTAT | 246 |
|  | R | AAGGTGGAAATGAGGCTGC |  |
| EGR2 | F | GACACGGCACATCCGAATC | 219 |
|  | R | GCACTGCTTTTCCGCTCTTT |  |
| HMGCR | F | CGCAACCTTTATATCCGTTTC | 271 |
|  | R | CCTCAATCATAGCCTCTGTGG |  |
| LPL | F | CTACAGAACAAAGAACGGCAT | 141 |
|  | R | AATAACTAACAAGGGTAGGGCT |  |
| CCL3 | F | GTGACCTCCACAGCTACCTCTTC | 245 |
|  | R | CTTTGGTGCCATGACTGCCTA |  |
| VCAM1 | F | AGTTGAAGGATGCGGGAG | 163 |
|  | R | ATGGCAGGTATTATTAAGGAGGA |  |
| HSPG2 | F | GGTTGCGGTGTGAGGAAGGT | 97 |
|  | R | TCGTGGTGTGTGTTGGTGAGG |  |
| ITGA1 | F | CATAATCCCTCGAAACACAACC | 236 |
|  | R | TGCCAAAGTAGGAACCAATCTG |  |
| TNC | F | GTGACAGAAGTGACGGAAGAGAC | 195 |
|  | R | GATGGCAAATACACGGATAAAGT |  |
| THBS1 | F | GGACATCCCAAAATGACCCTAAC | 217 |
|  | R | TTCCACATCACAACATAAAAGCG |  |
| GK | F | GAGGAATGACCAGCAACAAAAT | 147 |
|  | R | CGAGACTCCATACGCCGAC |  |
| PPARG | F | AGAAGGAGAAGCTGTTGGCG | 246 |
|  | R | GCAGGGGGGTGATGTGTTT |  |
| SLC27A1 | F | CTTTTGGGGACTGCAGGAAT | 267 |
|  | R | GGGGAAATGTGACAGGAACG |  |
| MMP1 | F | CGATTCGGGGAGAAGTGAT | 117 |
|  | R | GCCCATTTGGCAGTTGTG |  |
| PLTP | F | CATGCGGGATTCCTCACC | 220 |
|  | R | GAGGGGGCACTACAGGCTAT |  |

Abbreviations: F: forward; R:reverse.
