# Supplementary material for: Clinical significance and gene expression study of human hepatic stellate cells in HBV related-hepatocellular carcinoma
Source: J Exp Clin Cancer Res. 2013 Apr 19;32(1):22. doi: 10.1186/1756-9966-32-22 (PMC3654985; doi:10.1186/1756-9966-32-22)
Supplement: Additional file 2: Table S2 — Spearman rank correlation coefficient on all targets value. [file 1756-9966-32-22-S2.docx]

**Table S2. Spearman rank** **correlation coefficient on all targets value**

|  | A | | | P | | | Q | | | T | | |
| --- | --- | --- | --- | --- | --- | --- | --- | --- | --- | --- | --- | --- |
|  | A1 | A2 | A3 | P1 | P2 | P3 | Q1 | Q2 | Q3 | T1 | T2 | T3 |
| A1 | 1.000 | 0.995 | 0.941 | 0.782 | 0.772 | 0.674 | 0.804 | 0.772 | 0.894 | 0.769 | 0.662 | 0.768 |
| A2 | 0.995 | 1.000 | 0.944 | 0.771 | 0.762 | 0.664 | 0.802 | 0.766 | 0.897 | 0.759 | 0.656 | 0.762 |
| A3 | 0.941 | 0.944 | 1.000 | 0.723 | 0.694 | 0.592 | 0.737 | 0.685 | 0.942 | 0.731 | 0.594 | 0.714 |
| P1 | 0.782 | 0.771 | 0.723 | 1.000 | 0.921 | 0.869 | 0.873 | 0.848 | 0.719 | 0.931 | 0.828 | 0.882 |
| P2 | 0.772 | 0.762 | 0.694 | 0.921 | 1.000 | 0.917 | 0.849 | 0.849 | 0.681 | 0.848 | 0.864 | 0.891 |
| P3 | 0.674 | 0.664 | 0.592 | 0.869 | 0.917 | 1.000 | 0.816 | 0.817 | 0.611 | 0.808 | 0.843 | 0.919 |
| Q1 | 0.804 | 0.802 | 0.737 | 0.873 | 0.849 | 0.816 | 1.000 | 0.912 | 0.805 | 0.846 | 0.766 | 0.853 |
| Q2 | 0.772 | 0.766 | 0.685 | 0.848 | 0.849 | 0.817 | 0.912 | 1.000 | 0.734 | 0.800 | 0.791 | 0.811 |
| Q3 | 0.894 | 0.897 | 0.942 | 0.719 | 0.681 | 0.611 | 0.805 | 0.734 | 1.000 | 0.736 | 0.589 | 0.727 |
| T1 | 0.769 | 0.759 | 0.731 | 0.931 | 0.848 | 0.808 | 0.846 | 0.800 | 0.736 | 1.000 | 0.843 | 0.883 |
| T2 | 0.662 | 0.656 | 0.594 | 0.828 | 0.864 | 0.843 | 0.766 | 0.791 | 0.589 | 0.843 | 1.000 | 0.843 |
| T3 | 0.768 | 0.762 | 0.714 | 0.882 | 0.891 | 0.919 | 0.853 | 0.811 | 0.727 | 0.883 | 0.843 | 1.000 |
| A | 1 | | | 0.758 | | | 0.879 | | | 0.751 | | |
| P | 0.758 | | | 1 | | | 0.851 | | | 0.936 | | |
| Q | 0.879 | | | 0.851 | | | 1 | | | 0.839 | | |
| T | 0.751 | | | 0.936 | | | 0.839 | | | 1 | | |

Abbreviations: A: culture-activated hepatic stellate cells (HSCs); P: peritumoral HSCs; Q: quiescence phenotype HSCs; T: introtumoral myofibroblasts.
