# Supplementary material for: Clinical significance and gene expression study of human hepatic stellate cells in HBV related-hepatocellular carcinoma
Source: J Exp Clin Cancer Res. 2013 Apr 19;32(1):22. doi: 10.1186/1756-9966-32-22 (PMC3654985; doi:10.1186/1756-9966-32-22)
Supplement: Additional file 3: Table S3 — Representative genes in Gene Ontology analysis in different cell phenotypes. [file 1756-9966-32-22-S3.docx]

**Table S3 Representative genes in Gene Ontology** **analysis in different cell phenotypes (P<0.001)**

| **OG.ID** | **Term** | **Representative genes** |
| --- | --- | --- |
| 1. **Upregulated genes in peritumoral hepatic stellate cells (HSCs) vs quiescent HSCs** | | |
| **Biological Process** | | |
| 0010035 | response to inorganic substance | CAT/TNFRSF11B/UROS/TPM1/ PDGFA |
| 0007040 | lysosome organization | CLN3/PPT1/GBA/HEXB/ARSB/NAGPA/ACP2 |
| 0008610 | lipid biosynthetic process | PGAP2/PGAP3/ANG/PI4KA/PDGFA/PC |
| 0006066 | alcohol metabolic process | PPP1CA/SLC37A4/GPAT2/ENO2/PC/PGAM1/EBP |
| 0006629 | lipid metabolic process | SLC27A5/PGAP2/GPX1/PTPN11/CAT |
| 0007033 | vacuole organization | GBA/HEXB/ARSB/NAGPA/ACP2/CLN3/PPT1 |
| 0016126 | sterol biosynthetic process | EBP/DHCR24/MVK/SIGMAR1/DHCR7 |
| 0022603 | regulation of anatomical structure morphogenesis | TGFB1I1/TNFRSF11B/SERPINE1/TNFSF12/MBP |
| 0002541 | activation of plasma proteins involved in acute inflammatory response | SERPING1/F12/VSIG4/CFH/C2/ CD46/C1S |
| 0005975 | carbohydrate metabolic process | CHIT1/SLC37A4/BAD/ENO2/PC/ENO3 |
| 0044255 | cellular lipid metabolic process | SLC27A5/GAP2/GPX1/PTPN11/PI4KA/CERCAM/ |
| 0051346 | negative regulation of hydrolase activity | NEIL1/FKBP1B/GCHFR/ GPX1/SNCA/TP53 |
| **Cellular Component** | | |
| 0000323 | lytic vacuole | ARSG/DPP7/SIAE/SLC11A1/CAT/MMD/HLA-DMA |
| 0005764 | lysosome | C1ORF85/VAMP7/LGMN/RNASE2/SLC11A1/CAT |
| 0005773 | vacuole | CTSC/DPP7/CAT/C1ORF85/MMD/ SLC17A5 |
| 0005765 | lysosomal membrane | CLN3/ACP2/ MARCH9 |
| 0044437 | vacuolar part | SLC17A5/SLC48A1/LAPTM5/PLBD2/SAP |
| 0005774 | vacuolar membrane | C1ORF85/SLC48A1/MCOLN1/VAMP7/FAM176A |
| 0044444 | cytoplasmic part | ENO2/SIAE/SLC25A11/GSTK1 /PDGFA/VEGFC |
| **Molecular Function** | | |
| 0016798 | hydrolase activity, acting on glycosyl bonds | NTHL1/NAGPA/CHIT1/MAN1B1 |
| 0004553 | hydrolase activity, hydrolyzing O-glycosyl compounds | GBA/CHIT1/MAN1B1/EDEM2/HEXB |
| 0004522 | pancreatic ribonuclease activity | ANG/RNASE1/RNASE2/RNASE3/RNASE4 |
| 0016892 | endoribonuclease activity, producing 3'-phosphomonoesters | ANG/RNASE1/RNASE2/RNASE3/RNASE4 |
| 1. **Downregulated genes in peritumoral HSCs vs quiescent HSCs** | | |
| **Biological Process** | | |
| 0044260 | cellular macromolecule metabolic process | HMGB2/EGR1/UBR7/FBXW7/JUN/MAPK6/F4EBP2 |
| 0043170 | macromolecule metabolic process | IGHMBP2/TBRG1/RB1/GADD45A/CDK6/HBEGF |
| 0044237 | cellular metabolic process | ATG12/ATG5/E4F1/HMGB2 /FOXO3/PIK3C3 |
| 0006396 | RNA processing | TRA2B/SPOP/ POP/LCMT2/SRPK1/SMC1A/MRPL44 |
| 0008380 | RNA splicing | RBM5/SFRS4/TRA2B/PPIG/CIR1/RBM39 |
| 0016071 | mRNA metabolic process | DCP2/SMG1/DCPS/CPSF7SYMPK/SFRS17A |
| 0044238 | primary metabolic process | TBRG1/RB1/TAF1/DR1/EGR1/PS72 |
| 0010467 | gene expression | RB1/TAF1/ TBRG1/NAPG/IL1B/TLR2 |
| 0008152 | metabolic process | GLRX2/MED21/IGF1R/SP1/GNPNAT1/NAT15 |
| 0006397 | mRNA processing | RBM5/SF3A1/SF3A3/ WBP4/JMJD6/SYNCRIP |
| 0044267 | cellular protein metabolic process | CDC16/SOCS3/TRIM41/ATG12/PTPN2/PSMC1 |
| 0046907 | intracellular transport | OPTN/NPM1/ERGIC1/ATL2/JUN |
| 0009987 | cellular process | GTPBP4/RNASEN/IL2RA/SLC2A1/SLC22A1 |
| 0015031 | protein transport | OPTN/NUP205/GCKR/TNF/ MVP |
| 0016070 | RNA metabolic process | RB1/TAF1/SF3B14/PPP1R8/RBM28/RNPC3 |
| 0045184 | establishment of protein localization | OPTN/TLR2/IL1B/IL1A/CARD8 |
| 0006139 | nucleobase, nucleoside, nucleotide and nucleic acid metabolic process | IGF2/SWAP70/NLRP3/IRAK2/MAMLD1/ LIN54 |
| 0033036 | macromolecule localization | OPTN/CRY2/ALS2/SMG1/TIMM13/GDI1 |
| 0019538 | protein metabolic process | AKT2/ABL2/PTEN/CDKN2A/WDR5 |
| 0065003 | macromolecular complex assembly | SNRPG/SF1/ IST3H3/BRF2/ TBP/MED30 |
| 0044265 | cellular macromolecule catabolic process | DCP2/DC16//OCS3/TRIM41/ |
| 0008104 | protein localization | OPTN/IL1A/CARD8/GAPVD1/TAF8 /ALS2 |
| 0009057 | macromolecule catabolic process | DCP2 /TDG/IL1B/TNF/CST3/ERI1 |
| 0051649 | establishment of localization in cell | CCL3/BET1/SEC31A/ATL3/SEC22A/TMED10 |
| 0006807 | nitrogen compound metabolic process | CSF2/IGF1R/PDGFA/SP1/DNAJB6/ETS2 |
| 0048193 | Golgi vesicle transport | OPTN/GOLGA5/BET1/SEC24B/SEC24A/ATL2 |
| 0016265 | death | JUN/PMAIP1/BID/GGCT/CASP3/CASP7 |
| 0008219 | cell death | DYNLL1/TRAF1/CASP5/ ZAK/PIM1 |
| 0043933 | macromolecular complex subunit organization | RPL24/NUP205/RBM5/SMNDC1/SNRPE/SNRPG |
| 0006915 | apoptosis | TNFAIP8/GREM1/C3ORF38/SLC25A6/ IMP2 |
| 0006412 | translation | SELT/RPL10/RPL17/RPS26/HARS2/MARS |
| 0012501 | programmed cell death | AKAP13/NOTCH2/SGMS1/PIM1/TAF9 |
| 0016192 | vesicle-mediated transport | OPTN/CCL3/TMED10/AP4B1/ARFGAP2/AP3D1 |
| 0051641 | cellular localization | GOLGA5/STX1A/GOPC/ERGIC1/ATL2/GOSR2 |
| 0044085 | cellular component biogenesis | NPM1/NUP205/RBM5/SMNDC1/SNRPE/SNRPG/SF1 |
| 0006457 | protein folding | GLRX2/ERO1LB/FKBP1A/HSP90AA1/PFDN4/PPIA |
| 0048519 | negative regulation of biological process | TULP3/NLK/DCUN1D3 /CIR1/MSH6/ IL15/PLXNA3 |
| 0006888 | ER to Golgi vesicle-mediated transport | SCFD1 /EC24B/SEC24A/SEC31A |
| 0048523 | negative regulation of cellular process | DR1/EGR1/ZHX2/ZNF345/HSBP1/ PLXNA3 |
| 0019941 | modification-dependent protein catabolic process | UBR7/ SUMO2/TNFAIP3 /TRIM25/SOCS3 |
| 0043632 | modification-dependent macromolecule catabolic process | MKRN2/RNF144B/CUL5/HSP90B1 /TAF1 |
| 0009059 | macromolecule biosynthetic process | /IL1A/TLR6/MAMLD1/PDGFA/ HBEGF |
| 0051603 | proteolysis involved in cellular protein catabolic process | FEM1B/RNF139/TAF1/IL1B/TNF |
| 0044257 | cellular protein catabolic process | FEM1B/RNF139/MARCH3/RFFL/USP16 |
| 0032446 | protein modification by small protein conjugation | /MIB1/UBE2D3/UBE2H/CBLL1/CDKN2A |
| 0006461 | protein complex assembly | TUBG1/TSC1 /VMA21 /TAP2 /WASL |
| 0070271 | protein complex biogenesis | TAF1/USP16/SEPT9/BIRC3/TSC1/YWHAB |
| 0034645 | cellular macromolecule biosynthetic process | HMGB2/AKT2/CSF2/IL1B/TLR2 |
| 0000375 | RNA splicing, via transesterification reactions | RBM5/SF3A3/HNRNPC/TRA2B/ |
| 0016567 | protein ubiquitination | UBE2V1/RNF144B/RNF139/GTPBP4/MD2 |
| 0070647 | protein modification by small protein conjugation or removal | UBE2V1/SIAH2/UBE2D3/UBE2H/TSC1 |
| 0001775 | cell activation | IL15/TICAM1/SBNO2/TLR6/TLR2/ |
| 0044092 | negative regulation of molecular function | IRAK2/IL1B/PSMA6 /CDC16 |
| 0031396 | regulation of protein ubiquitination | GTPBP4/TSC1 /FKBP1A/PSMD14//CDC27 |
| 0022607 | cellular component assembly | NRCAM/DIAPH1/PDGFA/ VMA21/SACS |
| 0045321 | leukocyte activation | IL15/TICAM1/ TLR6/TLR2 /TNFSF14 |
| 0006367 | transcription initiation from RNA polymerase II promoter | TAF1/GTF2F2/TAF9/TAF12/TBP |
| 0031397 | negative regulation of protein ubiquitination | PSMD14/FZR1/CDC16/CDC27/ GTPBP4 |
| 0006352 | transcription initiation | TAF7/TAF9/ MED30/MED14/MED17 |
| 0030163 | protein catabolic process | FEM1B/RNF139/IL1B/TNF |
| 0044419 | interspecies interaction between organisms | RRAGA/TARDBP/TLR6/TLR2/NXF1/TRIM25 |
| 0006886 | intracellular protein transport | OPTN/NUP205/GCKR/TNF/NFKBIE |
| 0006366 | transcription from RNA polymerase II promoter | RB1/TAF1/DR1/EGR1 /JUN/CREB5 |
| 0006368 | RNA elongation from RNA polymerase II promoter | GTF2A2/ GTF2H1/POLR2D/POLR2E |
| 0000278 | mitotic cell cycle | TAF1/CDC123/CDKN1B/CDKN2A /SKP1 |
| 0000377 | RNA splicing, via transesterification reactions with bulged adenosine as nucleophile | RBM5/SMNDC1/SNRPE /SF1/ SLU7 |
| 0000398 | nuclear mRNA splicing, via spliceosome | RBM5/SMNDC1/ SNRPG/SF1/PM1 |
| 0031400 | negative regulation of protein modification process | FAM129A/IBTK/GTPBP4/TSC1//TAF7 |
| 0007049 | cell cycle | CASP3/DCUN1D3/SMARCA4/ SMD14 |
| 0033365 | protein localization in organelle | OPTN /TNF/NFKBIE/SNUPN/ |
| 0022613 | ribonucleoprotein complex biogenesis | GTPBP4/RNASEN/RPL24/ RBM5 /TSR1 |
| 0050866 | negative regulation of cell activation | PDGFA//CASP3/CD274/IL2RA |
| 0034613 | cellular protein localization | OPTN/NUP205/TNF/NFKBIE/SNUPN |
| 0051090 | regulation of transcription factor activity | CDKN2A/IRAK2/TRIB1/HMOX1/ID2 |
| 0090046 | regulation of transcription regulator activity | CDKN2A/ /IRAK2/TRIB1 /TLR2 |
| 0070727 | cellular macromolecule localization | OPTN/ /GCKR/TNF/NFKBIE/IL1B |
| 0016043 | cellular component organization | RPL24 /ATG12/ATG5/NUP205 /SF1/SNRNP200 |
| 0044249 | cellular biosynthetic process | CIR1/RBM39/BZW1/ZNF646/OXSM |
| 0009058 | biosynthetic process | E4F1/KIN/DNAJC2/HMGB2/IGHMBP2 |
| 0006354 | RNA elongation | GTF2A2/GTF2F2/GTF2H1 /POLR2E/ ELL |
| 0006916 | anti-apoptosis | BNIP3L/TNFAIP8/DUSP1/ERC1/ GF1R |
| 0016197 | endosome transport | VPS13A/ADRB2/RHOB/SNX1/FAM160A2 |
| 0002695 | negative regulation of leukocyte activation | CDKN2A/ /CD274/IL2RA/IL20RB/CD276/ |
| 0043086 | negative regulation of catalytic activity | DUSP6/ZFYVE28/TRIB1/FGFR1OP/CDKN2A |
| 0060255 | regulation of macromolecule metabolic process | RB1 /DR1/EGR1/SYNCRIP/HNRNPU |
| 0046649 | lymphocyte activation | ELF4/IL15/LRRC8A/SLAMF7/CD276//IL2RA |
| 0050868 | negative regulation of T cell activation | CD274/IL2RA/IL20RB/CD276 |
| 0051726 | regulation of cell cycle | CDKN2A/SMC1A/NBN/BCCIP/PTEN/IL1B |
| 0051438 | regulation of ubiquitin-protein ligase activity | PSMD14/FZR1/PSMA2/PSMA3/PSMA6/PSMB4 |
| 0043687 | post-translational protein modification | GLRX2/ERO1LB/ TLR6/BRD1/TAF9/CDC16 |
| 0031399 | regulation of protein modification process | APOA1/SOCS3/ /IL1B/BMP2/RNF139 |
| 0044248 | cellular catabolic process | ATG12/ATG5/DCP2/SMG1/IRG1/HSP90B1 |
| 0051437 | positive regulation of ubiquitin-protein ligase activity during mitotic cell cycle | PSMD14/FZR1/PSMA3 /CDC16//CDC27 |
| 0019222 | regulation of metabolic process | CDKN1B/CKS1B/GADD45A/GTPBP4 /PTEN/ |
| 0051250 | negative regulation of lymphocyte activation | CASP3/CD274/IL2RA/IL20RB/CD276 |
| 0051340 | regulation of ligase activity | PSMD14/FZR1/PSMA2 /PSMA6/PSMB4 |
| 0051352 | negative regulation of ligase activity | PSMD14/FZR1/PSMA2/CDC16/CDC27 |
| 0051444 | negative regulation of ubiquitin-protein ligase activity | PSMD14/FZR1/PSMA2/ CDC27/CDKN2A |
| 0048522 | positive regulation of cellular process | FAM129A/IL1B/TNF/F3/ CASP3//TRAF1 |
| 0022618 | ribonucleoprotein complex assembly | RPL24/RBM5/SMNDC1/SNRPE/SNRPG |
| 0009892 | negative regulation of metabolic process | DR1/EGR1/CD276/DNAJB6/CIR1/MSH6 |
| 0042981 | regulation of apoptosis | DAD1//GNRH1/ANXA1/BIRC3/XIAP/TAF9 |
| 0010941 | regulation of cell death | DAD1/F3/BID/CASP3/CUL5/MNT/P2RX4 |
| 0043412 | macromolecule modification | GLRX2/ERO1LB/GADD45B/ZAK/DRD4 |
| 0031324 | negative regulation of cellular metabolic process | EGR1/ZHX2/HIC2//RBPJ/GATAD2A |
| 0051439 | regulation of ubiquitin-protein ligase activity during mitotic cell cycle | PSMD14/FZR1/PSMA2/PSMA3/ PSMB4 |
| 0051443 | positive regulation of ubiquitin-protein ligase activity | PSMD14/FZR1/PSMD4/SKP1/CDC16 |
| 0048518 | positive regulation of biological process | FAM129A/ TNF/F3/ELF4/MMP12 |
| 0051246 | regulation of protein metabolic process | APOA1/SOCS3/FAM129A/IBTK |
| **Cellular Component** | | |
| 0044424 | intracellular part | NRIP1/CIR1/TAF9/KAT2B/FAM160A2 |
| 0005622 | intracellular | DNAJC19/CPT1A/DOCK4/PLXNA3 |
| 0043226 | organelle | CCDC90B/BID/TFB2M/MRPL14/MIB1 |
| 0043229 | intracellular organelle | SGMS1 /SEC24B/TUBB2A/TUBB6 |
| 0043227 | membrane-bounded organelle | SQLE/SRD5A1/HSPA13/TLR6/TLR1/PDGFA |
| 0043231 | intracellular membrane-bounded organelle | TAF1A/TAF9/KAT2B/BRF1/ TULP3 |
| 0070013 | intracellular organelle lumen | CDC5L/KIAA0020/CS/DLST/ALAS1 |
| 0031974 | membrane-enclosed lumen | NSUN2/RBM28/ELP3/ELP2/TSR1/UTP6 |
| 0043233 | organelle lumen | TAF7/MMS19 /TFB2M/SSBP1/BRD1 |
| 0005737 | cytoplasm | GOLGA5/SGMS1 /BET1/CDC123 |
| 0044446 | intracellular organelle part | BOD1/TEP1/TERF1/RSAD2/WDR5 |
| 0044422 | organelle part | RPA2/RPS6KA3/SKP1/SNRPD3/COL4A3BP |
| 0044428 | nuclear part | TBP/TDG/ING3/EZH2/BRD1 |
| 0031981 | nuclear lumen | THAP1/METTL3/ RB1/RPA2/ING3 |
| 0030529 | ribonucleoprotein complex | RPL10L/RPL10/RPL21/RPL24/RPL31/RPL39 |
| 0005634 | nucleus | NRIP1/CIR1/TAF1A/DK/LIN54/ZNF646 |
| 0005654 | nucleoplasm | PAX8/SYMPK/HIST3H3/CASP3/ CDC27 |
| 0032991 | macromolecular complex | SERP1 /BCCIP/ALS2/ DCAF6 |
| 0044444 | cytoplasmic part | PRKCI/GOLGA5 /CNIH/BET1/DLL1 |
| 0044451 | nucleoplasm part | SHFM1 /EZH2/BRD8/BRD1/WDR5 |
| 0005730 | nucleolus | MSN/ATF3 /NBN/THRAP3 |
| 0005681 | spliceosomal complex | SF3A1/RNPC3/SNRNP200/HNRNPU |
| 0043228 | non-membrane-bounded organelle | TAF1A/RPL10L/RPL10/PDE4DIP/ABI1 |
| 0043232 | intracellular non-membrane-bounded organelle | TAF1A /RPL10/RPL21/DYNLT3/KRTAP3-2 |
| 0016607 | nuclear speck | SMNDC1 /SF3A3/NOC3L/RBM8A |
| 0016604 | nuclear body | FBL/SRRM2/GAR1/U2AF1 |
| 0005840 | ribosome | RPL10/RPL21/RPL24/RPS18/MRPL51 |
| 0005829 | cytosol | ODC1/ODF2/FZR1/POMP/CDC42 |
| 0044464 | cell part | CENPN/NUP85/NDEL1/MYL12B/CCDC55 |
| 0005623 | cell | GJC1/ SPRY2/TLR6/FKBP11//ZNF830 |
| 0033279 | ribosomal subunit | RPL10L/HBA2/RPS8/RPS28/MRPL51/MRPL22 |
| 0016591 | DNA-directed RNA polymerase II, holoenzyme | POLR2D/TAF8/TAF1/GTF2H1/ INTS9 |
| 0043234 | protein complex | NRIP1/CIR1/TAF1A/CACYBP/ALS2/BMP2 |
| 0005794 | Golgi apparatus | GOLGA5/SGMS1/CNIH/BET1/ PRNP |
| 0012505 | endomembrane system | MEST/NSDHL /TLR6/TLR1/CDC42EP2 |
| 0015935 | small ribosomal subunit | HBA2/RPS8/RPS10/RPS12//DAP3 |
| 0016592 | mediator complex | MED19 /MED21/MED17/MED7 /THRAP3 |
| 0000502 | proteasome complex | PSMD1/PSMD2/PSMB4/PSMA2/ |
| 0005793 | ER-Golgi intermediate compartment | VMA21/ERGIC1/SURF4/TMEM49/YIF1A |
| 0005643 | nuclear pore | SEC13/NUP85 /SNUPN/NXF1/NUP50 |
| 0044445 | cytosolic part | RPL10/RPL21/RPL24/RPL31/CT4 |
| 0005739 | mitochondrion | ATP5E/ATP5L2/BNIP3L/TOMM34/CPT1A |
| 0046930 | pore complex | SNUPN/NXF1/NUP50/NUP205 |
| 0022626 | cytosolic ribosome | RPL10L/RPL10/RPL21 /RPL31/RPL39 |
| 0000139 | Golgi membrane | COPA/AFTPH /SGMS1/ST6GAL1/GOSR2 |
| **Molecular Function** | |  |
| 0005515 | protein binding | DNAJA2/SPAG9/TRIM41/CD59/CD97 |
| 0003723 | RNA binding | IGHMBP2/RPL21/SAFB/SNRPG/SF1/RBM4B |
| 0016251 | general RNA polymerase II transcription factor activity | MED19/CNOT2/MED15/MED20/TAF12 |
| 0008134 | transcription factor binding | RAD54L2/OASL/CALCOCO1/JUN/NBN |
| 0003712 | transcription cofactor activity | USP16/E4F1 /SND1/TFB2M/TMF1/PIR |
| 0005488 | binding | DNAJA2/GADD45B/ UGDH/PSME4/HEATR5A |
| 0003735 | structural constituent of ribosome | RPL10L/MRPL22/RPL39/MRPS5/RPL23/MRPL33 |
| 0016455 | RNA polymerase II transcription mediator activity | MED19/CNOT2/MED15/MED17/THRAP3 |
| 0003702 | RNA polymerase II transcription factor activity | ZNF345/IRF7 /ZNF143/ MED19 /HTATSF1 |
| 0003676 | nucleic acid binding | HNRNPK/HNRNPU/TEP1/RPF1/CALCOCO1 |
| 0000166 | nucleotide binding | AKT3/RIT1/ATL2/ /SFRS4/TRA2B |
| 0019210 | kinase inhibitor activity | TRIB1/CDKN1B/GMFB/PRKRIP1/SOCS3 |
| 0004860 | protein kinase inhibitor activity | CDKN1B/TRIB1/GMFB/PRKRIP1/SOCS3 |
| 0003713 | transcription coactivator activity | CREM/DCAF6/CALCOCO1/MED30/GTF2A2 |
| **Upregulated genes in intratumoral cancer-associated myofibroblasts (CAMFs) vs quiescent HSCs** | | |
| **Biological Process** | |  |
| 0007155 | cell adhesion | THRA/CDH2/CD209/COL12A1/VCAN/ |
| 0022610 | biological adhesion | VCL /NRP1/NPTN/ADAM10/ERBB3/TPM1 |
| 0048731 | system development | SMPD1/ TUBB2A/FZD2/CNFN/PRDX2 |
| 0007275 | multicellular organismal development | GDF11/TNC//GFBP7/PRDX2/DDAH1/NR2F6 |
| 0048856 | anatomical structure development | KAT2A/PRKDC/NRG1/KIF2A/RPS19/ |
| 0009653 | anatomical structure morphogenesis | PDPN /COL4A1/TPM1/PLAT/KRT18 |
| 0001944 | vasculature development | COL4A1 /ANGPTL6 /VEGFA/MMP2 |
| 0032502 | developmental process | NGFRAP1 /CHST3/COL4A2/TPM1/ ACP5 |
| 0001568 | blood vessel development | SHB/ CYR61/VEGFA/NRP1/SEMA5A |
| 0016477 | cell migration | SMO/CDK5/TWIST1/CCKAR/ROBO1 |
| 0006928 | cellular component movement | VEGFA/GREM1/DKK1/VNN2/CD9 |
| 0048870 | cell motility | SMO/CDK5/TWIST1/CXCL12 |
| 0051674 | localization of cell | MIA3/BDKRB1/VEGFA/GREM1/ADAM10 |
| 0051270 | regulation of cellular component movement | MIA3/BDKRB1/VEGFA/GREM1/CXCL12 |
| 0001935 | endothelial cell proliferation | ATP5A1/THBS1/CAV1/CAV2/ATPIF1 |
| 0001936 | regulation of endothelial cell proliferation | ATP5A1/THBS1 /CAV2/ATPIF1/CCL26 |
| 0002685 | regulation of leukocyte migration | MIA3/BDKRB1/VEGFA//CXCL12 |
| 0002687 | positive regulation of leukocyte migration | THBS1/ADAM10/CXCL12/BDKRB1 |
| 0040012 | regulation of locomotion | MIA3/BDKRB1/VEGFA/SCARB1/SNCA |
| 0043062 | extracellular structure organization | CDH2/MYO6 /TNC/ TGFBI /COL18A1 |
| 0030198 | extracellular matrix organization | COL1A2/COL3A1/COL5A1 /CRISPLD2 |
| 0055114 | oxidation reduction | NDUFB7/UQCRQ/FLJ44606/CYBRD1/FADS2/1 |
| 0030334 | regulation of cell migration | MIA3/BDKRB1/VEGFA /CDK5/LAMA4 |
| 0001525 | angiogenesis | CYR61/NRP1/SEMA5A/THBS1/VEGFC |
| 0048514 | blood vessel morphogenesis | COL4A1/ ANG/ANGPT1/TNFRSF12A/PLAT |
| 0001501 | skeletal system development | THRA /COL5A2 /MMP2/HSPG2/TWIST1 |
| 0051272 | positive regulation of cellular component movement | MIA3/BDKRB1 /THBS1/CXCL12 |
| 0048513 | organ development | THRA/COL4A1/ELK3/TUFT1/FZD2/CCKAR |
| 0000904 | cell morphogenesis involved in differentiation | S100A4/COL18A1/ /ERBB3/PARD3/PTPN11 |
| 0009611 | response to wounding | C3/PRDX2/F12/THBS1/IL6ST/OSMR |
| 0040011 | locomotion | SMO /TWIST1/CCKAR/ROBO1/MIA3 |
| 0060348 | bone development | FSTL3/COL5A2/EXT1/EXT2/SPARC/ |
| 0007409 | axonogenesis | SPON2/UNC5B/NTF3/ROBO1/NRP1/CCKAR |
| 0032535 | regulation of cellular component size | FHL1/NUPR1/IGFBP6/LIMA1/LAMB2 |
| 0006936 | muscle contraction | TNNT1/MYL9/ANXA6 /TPM1/VCL |
| 0003012 | muscle system process | TNNT1/CRYAB/ANKRD2/ITGB5/ASPH |
| 0010035 | response to inorganic substance | SERPINE1/PRKCA/CUTA/FXN/CAV1 |
| 0008283 | cell proliferation | SBDS/SCARB1/ATP5A1/THBS1/PRDX2 |
| 0009605 | response to external stimulus | COL3A1/CD9/SBDS /ERBB3/TPM1 |
| 0000271 | polysaccharide biosynthetic process | GBE1 /CERCAM/ALG1/CHST3/CHST12 |
| 0040017 | positive regulation of locomotion | MIA3/BDKRB1/SCARB1/PRKCA/CXCL12 |
| 0031175 | neuron projection development | CDK5/ERBB3/PARD3/SEMA5A/UCHL1 |
| 0048812 | neuron projection morphogenesis | CDK5/ERBB3/PARD3/PTPN11/NRP1 |
| 0001937 | negative regulation of endothelial cell proliferation | ATP5A1/THBS1/CAV1/CAV2/ATPIF1 |
| 0007399 | nervous system development | CDK5/TWIST1/CCKAR/VCAN/KCTD11 |
| 0018130 | heterocycle biosynthetic process | MTHFD1/PYCR1/ALDH18A1/MOCOS/UROS |
| 0032879 | regulation of localization | SNCA/MIA3/BDKRB1/GREM1/JPH2/CXCL12 |
| 0042221 | response to chemical stimulus | SERPINE1/PRKCA/KCNMB1/PDPN |
| 0043392 | negative regulation of DNA binding | COMMD7/RPS3/PRDX2/HDAC2/ |
| 0051128 | regulation of cellular component organization | GSN/TMSB4X/TMSL3/RAB3A/COL5A1 |
| 0048646 | anatomical structure formation involved in morphogenesis | COL4A1/ELK3/ANG/ANGPT1/MMP2 |
| 0031099 | regeneration | NEFL/VCAN/GSN/SERPINE1/TIMP3 |
| 0030335 | positive regulation of cell migration | MIA3/BDKRB1/VEGFA/SCARB1 /COL18A1 |
| 0016043 | cellular component organization | VCAN/CRYAB/LAMB1/EIF4EBP1/ALDOC |
| 0090066 | regulation of anatomical structure size | NUPR1/IGFBP6/IP6K2/NEFL/TNFRSF12A |
| 0006950 | response to stress | CTSD/CHEK1 /TPM1/SCAMP5/COL5A1 |
| 0001503 | ossification | FHL2/WWTR1/SMO/MMP2/5A2/EXT2 |
| 0048667 | cell morphogenesis involved in neuron differentiation | CDK5 /PARD3/TNFRSF12A/CDH2 |
| 0048468 | cell development | ANG/NPTN/LAMB2/CAV2/MSI2/ |
| 0007052 | mitotic spindle organization | KIF23/SBDS/RCC1/KIF11/TNKS |
| **Cellular Component** | | |
| 0031012 | extracellular matrix | MFAP4/COL18A1/VCAN/MMP2/TGFBI/ |
| 0044420 | extracellular matrix part | MFAP4/COL1A2/COL18A1/ COL6A1/TIMP3 |
| 0044421 | extracellular region part | MFAP4/ COL3A1/SPINT2/NOV/CRISPLD2 |
| 0005578 | proteinaceous extracellular matrix | MFAP4/COL1A2/COL3A1/TGFBI/PXDN |
| 0044444 | cytoplasmic part | ENO2/ITPR1/PTGDS/TRPC4/ANXA6 |
| 0005576 | extracellular region | MFAP4/COL1A2/PTX3/SCPEP1/FAM3A |
| 0005581 | collagen | COL1A2/COL3A1/COL10A1/COL18A1 |
| 0005737 | cytoplasm | ATPIF1/PLP2/ALG1/RHBG/TRPC4 |
| 0005604 | basement membrane | COL4A1/COL4A2/COL4A4/ANG |
| 0044449 | contractile fiber part | MYL9/TNNT1/TPM1/ MMP2/VCL |
| 0005739 | mitochondrion | ATP5A1/HINT2/RTN4IP1/PIGY/CAV1 |
| 0043292 | contractile fiber | MYL9/MYL6B/TNNT1/TPM1/TPM2/ VCL |
| 0005615 | extracellular space | THBS1/IGFBP3/ ANGPTL6/ATRN/EMR3 |
| 0005605 | basal lamina | LAMA4/LAMB1/LAMB2/ANG/HSPG2 |
| 0032432 | actin filament bundle | TPM1/PSTPIP1/PDLIM7/SEPT7/CRYAB |
| 0042641 | actomyosin | LIMA1/TPM1/PSTPIP1/PDLIM7/SEPT7 |
| **Molecular Function** | |  |
| 0005201 | extracellular matrix structural constituent | COL12A1/TUFT1/COL1A2/COL3A1/COL4A1 |
| 0019838 | growth factor binding | IL6ST/NTF3/IGFBP4/IGFBP7/CYR61 |
| 0005198 | structural molecule activity | RPL39L/RPL17/VCL/TSGA10/ SEPT7 |
| 0005515 | protein binding | CRYAB/PPIC/VAMP7/EPT7/NUAK1 |
| 0016706 | oxidoreductase activity, acting on paired donors, with incorporation or reduction of molecular oxygen, 2-oxoglutarate as one donor, and incorporation of one atom each of oxygen into both donors | ASPHD1/ASPH/P4HA2/PLOD2/ALKBH3 |
| 0048407 | platelet-derived growth factor binding | COL1A2/COL3A1/COL4A1/COL5A1 |
| 0005520 | insulin-like growth factor binding | IGFBP3/IGFBP6/IGFBP4/IGFBP7/CYR61/NOV |
| 0016491 | oxidoreductase activity | CYBRD1/MSRB3/HSD17B10 /HEPH |
| 0031418 | L-ascorbic acid binding | ALKBH3/PAM/PLOD2/LEPRE1/P4HA2 |
| 0042802 | identical protein binding | ADAM10/CEBPE/SNCA/THBS1/CDC42BPA/CAV1 |
| 1. **Downregulated genes in intratumoral CAMFs vs quiescent HSCs** | | |
| **Biological Process** | | |
| 0002376 | immune system process | CASP3/ELF4 /ID2/IRF1/DEFB1/TRIM25/MARCO |
| 0043170 | macromolecule metabolic process | MLH1/E4F1/IGHMBP2/NUP62/ADRB2 |
| 0019915 | lipid storage | EHD1/NR1H3/ABCA1/PPARA/PPARG |
| 0044237 | cellular metabolic process | MLH1/PSEN1/MAP1LC3A/ATG12/DND1/TGDS |
| 0008152 | metabolic process | PSEN1/MAP1LC3A/ARID3B/NEU4/ENPP4 |
| 0010745 | negative regulation of macrophage derived foam cell differentiation | NR1H3/CETP/ABCA1/ITGB3/PPARA |
| 0044260 | cellular macromolecule metabolic process | MLH1/E4F1/IGHMBP2/PAX8/ZNF655/FAM120B |
| 0044238 | primary metabolic process | MLH1/E4F1 /GPR109B/TICAM1/OXSM/DND1 |
| 0010878 | cholesterol storage | EHD1/NR1H3/ABCA1/PPARA/PPARG |
| 0006955 | immune response | TICAM1/SBNO2/TLR2/IGLL1/IL1A/IL16 |
| 0048518 | positive regulation of biological process | IL1B/ITLN1/PSEN1/TNF/MED17/PSMD4 |
| 0007242 | intracellular signaling cascade | FGF13/RAPGEF2/MAP3K4/ADA/P2RX4 |
| 0010885 | regulation of cholesterol storage | EHD1/NR1H3/ABCA1/PPARA/PPARG/ABCG1 |
| 0010888 | negative regulation of lipid storage | NR1H3/ABCA1/PPARA/PPARG/ABCG1/ITGB3 |
| 0019538 | protein metabolic process | MMP1/MMP3/MMP10/NR1H3/WDR5 |
| 0044267 | cellular protein metabolic process | MAP3K4/ZAK/PSEN1/DRD4/IL1B/ TSC1 |
| 0016265 | death | MNT/P2RX4/TRAF1/DCUN1D3/ADRB2 |
| 0010876 | lipid localization | OSBPL8/STARD5/ABCG1/IL1B//TNF |
| 0008380 | RNA splicing | SNRPG/SF1/SNRNP200/BCAS2/TRA2B |
| 0008219 | cell death | BID/GGCT/CASP3/CASP7/SPG20/TARDBP/ |
| 0048519 | negative regulation of biological process | MLH1/ZHX2/YRDC/PSMD14/CDC26/ |
| 0046907 | intracellular transport | GCKR/TN/ALS2/VPS4B/SFRS13B/PEX26 |
| 0010742 | macrophage derived foam cell differentiation | CETP/ABCA1/ITGB3/PPARA/PPARG |
| 0090077 | foam cell differentiation | SOAT1/NR1H3/CETP/ABCA1/ITGB3/PPARA |
| 0002437 | inflammatory response to antigenic stimulus | IL20RB/IL1B/TNF/CD24/IL2RA |
| 0048523 | negative regulation of cellular process | ZHX2/ZNF345/KANK1 /MAPRE1 |
| 0010883 | regulation of lipid storage | EHD1/NR1H3/ABCA1/PPARA/PPARG/ABCG1 |
| 0008285 | negative regulation of cell proliferation | CDKN2A/CASP3/CD274/IL2RA/IL20RB |
| 0008637 | apoptotic mitochondrial changes | JUN/BID/GGCT/CASP3/CASP7/CD24 |
| 0002237 | response to molecule of bacterial origin | TLR2/TRIB1/ADM/TICAM1/CYP27B1 |
| 0048522 | positive regulation of cellular process | IL1B /PSEN1/TNF/ PSMD14/SKP1 |
| 0002520 | immune system development | PLEK/PSEN1/ADA/IL15/RUNX1//MJD6 |
| 0012501 | programmed cell death | JUN/BID/GGCT/CASP3/BTG2/PLCG2 |
| 0006954 | inflammatory response | IL1A/IL1B//TNF/CD24/CCL4/CCL20/CCL23 |
| 0006915 | apoptosis | PPP3CC/YWHAB/MNT/DUSP1/ZAK/ING3 |
| 0007243 | protein kinase cascade | FGF13/RAPGEF2/MAP3K4/OXSR1 |
| 0043065 | positive regulation of apoptosis | CDKN2A/NACC1/NOTCH2/ PTEN/BNIP3L/MAP3K5 |
| 0010743 | regulation of macrophage derived foam cell differentiation | NR1H3/CETP/ABCA1/ITGB3/PPARA/PPARG |
| 0043068 | positive regulation of programmed cell death | CD24/CDKN1B/CDKN2A/NACC1/NLRP3 |
| 0010942 | positive regulation of cell death | CD24/CDKN1B /NACC1/NLRP3/STK3 |
| 0034612 | response to tumor necrosis factor | CYP27B1/GCH1/CXCL16/CASP3/RIPK1 |
| 0009611 | response to wounding | IL1A/IL1B/IL20RB/IL2RA/TNF |
| 0042130 | negative regulation of T cell proliferation | CDKN2A/CASP3/ CBLB/CD274/IL2RA/IL20RB |
| 0006464 | protein modification process | MAP3K4/ZAK/RIMKLA/PCMTD2/RABGGTB |
| 0006793 | phosphorus metabolic process | CDKN1B/IL1B/TNF/DUSP6/TSC1/GNPTAB |
| 0006796 | phosphate metabolic process | DYRK3/RIOK3/PRPF4B/MAP3K14/TSC1 |
| 0006809 | nitric oxide biosynthetic process | TICAM1/IL1B/P2RX4/TNF/DDAH2 |
| 0006869 | lipid transport | AKT2/CETP/NR1H3/PPARA/PLIN2/DRD4 |
| 0050728 | negative regulation of inflammatory response | IL20RB/PPARG/ADA/ADRB2/IL2RA |
| 0015031 | protein transport | TNF/EPS15/RAB18/SNX8//RAB43 |
| 0045429 | positive regulation of nitric oxide biosynthetic process | TICAM1/HBB/HSP90AA1/IL1B/P2RX4/TNF |
| 0045321 | leukocyte activation | ELF4/IL15/ADA/ CASP3/PLDN/JMJD6/MLL5 |
| 0030097 | hemopoiesis | PLEK/PSEN1/ADA/PLCG2/CD24/NOTCH2 |
| 0046209 | nitric oxide metabolic process | DDAH2/GCH1/TICAM1/HBB/HSP90AA1 |
| 0033036 | macromolecule localization | GCKR/TNF/PCSK5/DUSP16/NUDT4/ PLEK |
| 0032496 | response to lipopolysaccharide | TRIB1/IL1B//UN /THBD/IRG1/SOCS3 |
| 0010608 | posttranscriptional regulation of gene expression | SELT/EIF2C4/EIF1B/CIRBP/DICER1/PTEN |
| 0001775 | cell activation | ELF4/IL15/TICAM1/SBNO2/TLR2/PSEN1 |
| 0016311 | dephosphorylation | CTDSP2/DUSP18/DUSP1/6/DUSP28/PDP1 |
| **Cellular Component** | | |
| 0005622 | intracellular | NRIP1/TAF1A/GTF3C6/ RAPGEF2/STARD8 |
| 0044424 | intracellular part | OXSM/ MMP3/KRTAP3-2/DCUN1D3 |
| 0005737 | cytoplasm | SGMS1/STX6/SEC24B/NEU4/CYTH1/TRIP4 |
| 0005829 | cytosol | RPL21/ UGP2/UPP1 /GGCT/CDC42 |
| 0043227 | membrane-bounded organelle | GTF3C6//GMS1/MMP3/ATG12/TNF/KIF5B |
| 0043231 | intracellular membrane-bounded organelle | GAK/MOBKL3/GOLGA1/PCSK5/RPGR/ST6GAL1 |
| 0005634 | nucleus | UPF3B/SUMO2/SDCCAG1 /SYAP1 |
| 0044428 | nuclear part | PPP1CB/CHFR/MLL5/YLPM1/ING3 |
| 0005625 | soluble fraction | ABI1/CTDSP2/TPP1/IL17C |
| 0031981 | nuclear lumen | NRIP1/TAF1A/GTF3C6 /SRRM2/U2AF1 |
| 0005681 | spliceosomal complex | RNPC3/HNRNPR/BCAS2//U2AF1/SF1 |
| 0044444 | cytoplasmic part | SGMS1/STX6/SEC24B/ST6GALNAC2/GTPBP4 |
| 0043226 | organelle | ACTR10/ACTR3B/SNTB1/TBCA/SPRY2 |
| 0043229 | intracellular organelle | MMP3/ATG12/KRTAP3-2/TNF |
| 0070013 | intracellular organelle lumen | ING3/MLL2/EZH2/NEU4/WDR5/C17ORF49 |
| **Molecular Function** | | |
| 0005515 | protein binding | COPA/SFRS13B/DNAJA4/RP2/TSC1 |
| 0008009 | chemokine activity | CXCL2/CXCL3/CCL3L3/CXCL16/CCL3/CCL4 |
| 0019210 | kinase inhibitor activity | TRIB1/CDKN1B/PKIB/SOCS3/CDKN2A |
| 0042379 | chemokine receptor binding | CXCL2/CXCL3/CCL3L3/CXCL16 |
| 0005488 | binding | DNAJA2/COPA/DHRS12/GFOD2/RGPD5 |
| 0016455 | RNA polymerase II transcription mediator activity | MED10/MED30/MED14/MED17/MED7 |
| 0004860 | protein kinase inhibitor activity | CDKN1B/CDKN2A/CASP3/PKIB/IBTK |
| 0016278 | lysine N-methyltransferase activity | MLL2/DOT1L/SETDB1/WDR5/MLL5 |
| 0016279 | protein-lysine N-methyltransferase activity | EZH2/PRDM2/MLL2/DOT1L/SETDB1/WDR5 |
| 0018024 | histone-lysine N-methyltransferase activity | MLL5/EZH1/EZH2/PRDM2/MLL2/DOT1L |
| 1. **Upregulated genes in peritumoral HSCs vs intratumoral CAMFs** | | |
| **Biological Process** | | |
| 0006952 | defense response | PPARG/SERPINA3/SERPINA1/DEFB1/MARCO |
| 0006954 | inflammatory response | SERPINA1/CFD/C2/C1QA/C1QB |
| 0006955 | immune response | SLC11A1 /CFD/C2/RB3 |
| 0009605 | response to external stimulus | CTSD/ TRPV4/SLC11A1/RBP4/IGFBP1 |
| 0009611 | response to wounding | WAS/FOS/FPR2/SLC11A1/C1QA/C1QB |
| 0002376 | immune system process | SLC11A1/ARRB2/SIRPG/RBP4 |
| 0031348 | negative regulation of defense response | PPARG/ARRB2/ADRB2/SERPINF1/TNFRSF1B |
| 0002682 | regulation of immune system process | SLC11A1/C1QA/C1QB/IL18/SPACA3 |
| 0009409 | response to cold | ADRB2/FOS/IL18/PPARG |
| 0006950 | response to stress | CTSD/CD24/SLC2A8/IL18/SERPINA1/TNFRSF1B |
| 0002526 | acute inflammatory response | PPARG/SERPINA3/SERPINA1/CFD |
| 0045087 | innate immune response | CFD/C2/SLC11A1/ARRB2/DEFB1/MARCO |
| 0002684 | positive regulation of immune system process | SLC11A1 /CFD/C2/C1QA/C1QB |
| 0002449 | lymphocyte mediated immunity | SLC11A1/ C1QA/C1QB/C2/ARRB2 |
| 0048583 | regulation of response to stimulus | PPARG/CD24/CD40/SLC11A1 |
| 0051180 | vitamin transport | SLC46A1/FOLR2/TCN2/RBP4 |
| 0002460 | adaptive immune response based on somatic recombination of immune receptors built from immunoglobulin superfamily domains | SLC11A1/CD24/C1QA/C1QB |
| 0002250 | adaptive immune response | SLC11A1/CD24/C1Q/C2//IL18 |
| 0002252 | immune effector process | SLC11A1/CD24/CFD/C2/C1QA |
| 0050728 | negative regulation of inflammatory response | PPARG/ADRB2//ERPINF1/TNFRSF1B |
| 0002443 | leukocyte mediated immunity | SLC11A1/CD24/C1QA/C1QB/C2/ARRB2 |
| 0050776 | regulation of immune response | CD24/CD40/C1QA/C1QB/ARRB2/RBP4 |
| 0006897 | endocytosis | ARRB2/ADRB2/HCK/VAV1/CD24 |
| 0010324 | membrane invagination | ARRB2/MARCH2/RIN3/ADRB2 |
| 0006956 | complement activation | CFD/C2/C1QA/C1QB |
| 0050727 | regulation of inflammatory response | PPARG/CD24/ADRB2/SERPINF1/TNFRSF1B |
| 0023052 | signaling | CTSD/KCNH1/PCSK5/C1QA/SLC2A8 |
| 0045730 | respiratory burst | NOXA1/CD24/SLC11A1 |
| 0002541 | activation of plasma proteins involved in acute inflammatory response | CFD/C2/C1QA/C1QB |
| 0032102 | negative regulation of response to external stimulus | PPARG/ADRB2/SERPINF1//NFRSF1B |
| 0050873 | brown fat cell differentiation | ADRB2/LAMB3/PPARG |
| 0055085 | transmembrane transport | SLC46A1/FOLR2/TCN2/RBP4/SLC24A6 |
| 0051605 | protein maturation by peptide bond cleavage | PCSK5/CFD/C2/C1QA/C1QB |
| 0050896 | response to stimulus | CTSD/FOS/CD24/SLC2A8/IL18/SLC24A6 |
| **Cellular Component** | | |
| 0000323 | lytic vacuole | SPACA3/CTSD /MAN2B1/CTSA |
| 0005764 | lysosome | HPSE/C1ORF85/GBA/MARCH2/ MAN2B1/ SLC11A1 |
| 0005773 | vacuole | SPACA3/SLC11A1/HPSE/C1ORF85/GBA/MARCH2 |
| 0031226 | intrinsic to plasma membrane | LILRB3/CNR1/ADRB2/EMR1/FCER1G |
| 0044459 | plasma membrane part | GNA15/LILRB3/CNR1/ADRB2/EMR1/FCER1G |
| 0005887 | integral to plasma membrane | NOXA1/LILRB3/CNR1/ADRB2/EMR1 |
| 0031982 | vesicle | SPACA3/PCSK5/SLC40A1/WAS/SERPINF1 |
| 0005765 | lysosomal membrane | HPSE/C1ORF85/GBA/MARCH2 |
| 0031988 | membrane-bounded vesicle | SPACA3/PCSK5/SLC40A1/WAS /ARRB2 |
| 0005886 | plasma membrane | GNA15/LILRB3/CNR1/PAM1/PPAP2B |
| 0031410 | cytoplasmic vesicle | SPACA3/PCSK5/SLC40A1/CFD/ RHCG |
| **Molecular Function** | | |
| 0008236 | serine-type peptidase activity | CFD/MMP8/PCSK5/C2/CPVL/CTSA |
| 0017171 | serine hydrolase activity | PCSK5/C2/CPVL/CTSA/DPP7 |
| 0030234 | enzyme regulator activity | RENBP/PKIB/SERPINA3/SERPINF1/GPSM3 |
| 0004867 | serine-type endopeptidase inhibitor activity | SERPINA3/SERPINF1/SERPINA1/SERPINB3/SERPINB4 |
| 0004553 | hydrolase activity, hydrolyzing O-glycosyl compounds | SPACA3/GBA/SPAM1/MAN2B1/HPSE |
| 0004866 | endopeptidase inhibitor activity | SERPINA3/SERPINF1/SERPINA1/SERPINB3 |
| 0030414 | peptidase inhibitor activity | RENBP/SERPINA3/SERPINF1/SERPINA1/SERPINB3 |
| 0005243 | gap junction channel activity | GJA3/GJB2 |
| 0016798 | hydrolase activity, acting on glycosyl bonds | SPACA3/GBA/SPAM1/MAN2B1/HPSE |
| 0022829 | wide pore channel activity | GJA3/GJB2 |
| 0051183 | vitamin transporter activity | SLC46A1/RBP4 |
| 0005542 | folic acid binding | SLC46A1/FOLR2 |
| 0004857 | enzyme inhibitor activity | PKIB/RENBP//ERPINA3/SERPINF1/SERPINA1 |
| 1. **Downregulated genes in peritumoral HSCs vs intratumoral CAMFs** | | |
| **Biological Process** | |  |
| 0030198 | extracellular matrix organization | COL1A2/SERPINH1/EMILIN1/RECK |
| 0043062 | extracellular structure organization | CDH2/PCDHB2/TNC/EMILIN1/COL4A2/ |
| GO:0007155 | cell adhesion | CDH2/AMIGO2/CDK6/FAT1/CTNNAL1 |
| 0022610 | biological adhesion | AMIGO2/CDK6/FERMT2/CYTH3/KIFAP3 |
| 0032502 | developmental process | SERPINE1/COL1A2/COL3A1/CAV2/ITGA1 |
| 0001568 | blood vessel development | COL4A1 /THY1/VEGFC /RECK/THBS1 |
| 0001944 | vasculature development | COL4A1/ELK3/THY1/VEGFC/COL18A1 |
| 0048856 | anatomical structure development | COL1A2/COL3A1/TUBB2A/HSD11B1/CAV2 |
| 0055114 | oxidation reduction | NDUFA9/STEAP1/FLJ44606/CYBRD1/ASPHD1 |
| 0016043 | cellular component organization | FBXO5/TUBA1B/ETS1/ARPC5L/CCL26/NEXN |
| 0048731 | system development | COL1A2/COL3A1/COL5A2/IGFBP3//TPM1 |
| 0030199 | collagen fibril organization | COL1A2/COL3A1/COL5A1/SERPINH1 |
| 0007275 | multicellular organismal development | COL1A2/COL3A1/SNAI1 /IGFBP3/S1PR5 |
| 0006936 | muscle contraction | TPM1/CNN3/CACNA1C /VCL |
| 0016477 | cell migration | PLAT/IGFBP3/LAMA4/SERPINE2/CLIC4 |
| 0048870 | cell motility | FYN/MDGA1/BDKRB1/GREM1/DKK1 |
| 0051674 | localization of cell | FYN/MDGA1/BDKRB1/GREM1/DKK1 |
| 0030334 | regulation of cell migration | /GREM1/THBS1/IGFBP3/CCL26/SERPINE2 |
| 0051270 | regulation of cellular component movement | BDKRB1/NEXN/CCL26 /THY1/ETS1 |
| 0003012 | muscle system process | GJC1/CRYAB/MYLK/ACTA2/TPM2 |
| 0061061 | muscle structure development | CRYAB/CSRP2/AEBP1/ITGA11/TAGLN |
| 0006221 | pyrimidine nucleotide biosynthetic process | UMPS/NME1/TYMS/DTYMK/CTPS |
| 0006928 | cellular component movement | FYN/MDGA1 /GLI2/NTF3/CTHRC1 |
| 0009653 | anatomical structure morphogenesis | COL4A1/ THY1/VEGFC/COL18A1/COL5A2 |
| 0008285 | negative regulation of cell proliferation | THBS1/CAV1/CAV2/IGFBP3/CDK6 |
| 0032964 | collagen biosynthetic process | COL3A1/COL5A1/SERPINH1/TRAM2 |
| 0040012 | regulation of locomotion | BDKRB1/GREM1/THBS1/IGFBP3/LAMA4 |
| 0045785 | positive regulation of cell adhesion | CDK6/EMILIN1/CYR61/THBS1/KIFAP3 |
| 0030336 | negative regulation of cell migration | GREM1/IGFBP3/ CLIC4/THY1/VCL |
| 0040013 | negative regulation of locomotion | GREM1/IGFBP3/CLIC4/THY1/TPM1/VCL |
| **Cellular Component** | | |
| 0031012 | extracellular matrix | MFAP4/COL1A2/ TNFRSF11B/TNC/SPARC/TIMP3 |
| 0005578 | proteinaceous extracellular matrix | MFAP4/COL1A2/COL3A1/COL4A1/ 2/TNC |
| 0044420 | extracellular matrix part | MFAP4/COL1A2/COL3A1/COL4A1/HSPG2 |
| 0005581 | collagen | COL1A2/COL3A1/COL4A1 |
| 0044444 | cytoplasmic part | BET1/CACNA1C/ SPARC/SEPT11 |
| 0044421 | extracellular region part | COL3A1/COL4A1/ S100A13/SOD3 |
| 0005737 | cytoplasm | RASGRP1/CNIH/BET1/SEC23A/CYTH3 |
| 0044449 | contractile fiber part | MYL9/TPM1/TPM2/TPM4/TRIM32 |
| 0005783 | endoplasmic reticulum | PDIA5/CERCAM/FKBP14/KIAA0020 |
| 0043292 | contractile fiber | MYL9/TPM4//TRIM32/SYNE1/PDLIM5 |
| 0005604 | basement membrane | COL4A1/COL4A2/HSPG2/COL18A1 |
| 0030016 | myofibril | TPM//TPM4/TRIM32/SYNE1/PDLIM5 |
| 0030017 | sarcomere | TPM1/TPM2/TPM4/TRIM32/PDLIM5 |
| 0005576 | extracellular region | MFAP4/COL1A2/COL3A1/COL4A1/COL4A2 |
| 0005912 | adherens junction | LMO7/TJP1/CDH2/VCL/NEXN |
| 0015629 | actin cytoskeleton | FERMT2/LIMA1/SEPT11/TPM1/MYL9 |
| 0005924 | cell-substrate adherens junction | FERMT2/FHL2/LIMA1/FBLIM1/LIMS2 |
| 0071212 | subsynaptic reticulum | CERCAM/FKBP14/RCN1/SEC31A/MOXD1 |
| 0044432 | endoplasmic reticulum part | PDIA5/CERCAM/FKBP14/RCN1/TXNDC5 |
| 0043228 | non-membrane-bounded organelle | RPL39L/RPL23A/RPSA/ACTA2/FSCN1 |
| 0043232 | intracellular non-membrane-bounded organelle | RPL39L/RPL23A/RPSA/ACTA2/KIFAP3 |
| 0030055 | cell-substrate junction | NEXN/FERMT2/FHL2/LIMA1/CAV1 |
| 0070161 | anchoring junction | LMO7/TJP1/NEXN/AFAP1/TGFB1I1 |
| 0005583 | fibrillar collagen | COL1A2/COL3A1/COL5A1/COL5A2 |
| 0005925 | focal adhesion | FERMT2/FHL2/LIMA1/FBLIM1/LIMS2 |
| 0032432 | actin filament bundle | FERMT2/LIMA1/SEPT11/TPM1/CRYAB |
| 0042641 | actomyosin | FERMT2/LIMA1/SEPT11/TPM1/DBN1 |
| 0005829 | cytosol | RPL39L/RPL23A/RPSA/RPS4Y1/BAG3 |
| 0030018 | Z disc | CRYAB/FHL2/JPH2/CACNA1C/SYNC |
| 0005788 | endoplasmic reticulum lumen | CALU/PDIA5/CERCAM/FKBP14/RCN1 |
| 0044424 | intracellular part | RASGRP1/CNIH/BET1/RAB12/CUL4B |
| **Molecular Function** | | |
| 0005201 | extracellular matrix structural constituent | EMILIN1 /COL3A1/COL4A1/COL4A2 |
| 0005198 | structural molecule activity | EMILIN1/MRPL3/RPL39L/RPL22L1/CTNNAL1 |
| 0016706 | oxidoreductase activity, acting on paired donors, with incorporation or reduction of molecular oxygen, 2-oxoglutarate as one donor, and incorporation of one atom each of oxygen into both donors | ASPHD1/ASPH/P4HA2/PLOD2/LEPREL2/ |
| 0031418 | L-ascorbic acid binding | LEPREL2/PAM/PLOD2/LEPREL1/OGFOD1/LEPRE1 |
| 0048407 | platelet-derived growth factor binding | COL1A2/COL3A1/COL4A1/COL5A1/COL6A1 |
| 0016491 | oxidoreductase activity | CYBRD1/MSRB3/NDUFA9/UGDH/ALDH7A1 |
| 0005515 | protein binding | CHAF1A/CCT2/GRPEL2/DNAL1/SRPX/RBX1 |
| 0005518 | collagen binding | THBS1/DCN/ITGA11/ITGA1/SPARC/ANTXR1 |
| 0016705 | oxidoreductase activity, acting on paired donors, with incorporation or reduction of molecular oxygen | PAM/ASPHD1/ASPH/P4HA2/JMJD6 |
| 0016702 | oxidoreductase activity, acting on single donors with incorporation of molecular oxygen, incorporation of two atoms of oxygen | LEPREL2/JMJD6/ASPHD1/ASPH |
| 0051213 | dioxygenase activity | LEPREL2/JMJD6/ASPHD1/ASPH |
| 0016701 | oxidoreductase activity, acting on single donors with incorporation of molecular oxygen | LEPREL2/JMJD6/ASPHD1/ASPH/PLOD2 |
| 0008092 | cytoskeletal protein binding | PDLIM5/SYNPO/CNN1/CNN3/DBN1 |
| 0019838 | growth factor binding | NTF3/IGFBP7/CYR61/THBS1/IGFBP3 |
| 0003779 | actin binding | LIMA1/FSCN1/TPM4/VCL/ARPC5L |
| 0005488 | binding | CHAF1A/CCT2/ SLC25A3/PYCR1/ARMCX2 |
| 0030246 | carbohydrate binding | PTX3/VCAN/LPHN2/GFPT1/CLEC2D |
| 0001871 | pattern binding | PTX3/DCN/VCAN/FSTL1/COL5A1 |
| 0030247 | polysaccharide binding | PTX3/DCN/VCAN/FSTL1/THBS1 |
| 0005509 | calcium ion binding | CDH2/RASGRP1/MYL9/FSTL1/VCAN |
