# Supplementary material for: Clinical significance and gene expression study of human hepatic stellate cells in HBV related-hepatocellular carcinoma
Source: J Exp Clin Cancer Res. 2013 Apr 19;32(1):22. doi: 10.1186/1756-9966-32-22 (PMC3654985; doi:10.1186/1756-9966-32-22)
Supplement: Additional file 4: Table S4 — Representative genes in pathway analysis in different cell phenotypes. [file 1756-9966-32-22-S4.docx]

**Table S4 Representative genes in pathway analysis in different cell phenotypes (P<0.001)**

| **PathwayID** | **Definition** | **Representative genes** |
| --- | --- | --- |
| 1. **Upregulated genes in peritumoral hepatic stellate cells (HSCs) vs quiescent HSCs** | | |
| hsa04142 | Lysosome | ACP2/CLN3/CTSF/CTSK/FUCA1/GBA/PPT1 |
| hsa00511 | Other glycan degradation | FUCA1/GBA/HEXA/HEXB/MAN2B1/NEU2 |
| hsa00604 | Glycosphingolipid biosynthesis - ganglio series | HEXA/HEXB/ST3GAL5/ST6GALNAC4 |
| hsa04610 | Complement and coagulation cascades | C1S/C2/C3/C3AR1/CD46/CFH/F12/SERPINE1 |
| hsa00100 | Steroid biosynthesis | C5ORF4/DHCR24/DHCR7/EBP |
| hsa00531 | Glycosaminoglycan degradation | ARSB/HEXA/HEXB/NAGLU |
| hsa04270 | Vascular smooth muscle contraction | ACTG2/GNA12/ITPR1/PLA2G4E/PPP1CA |
| hsa00520 | Amino sugar and nucleotide sugar metabolism | CHI3L1/CHIT1/GALE/HEXA/HEXB/UAP1L1 |
| hsa04978 | Mineral absorption | ATP1A4/CYBRD1/FXYD2/HEPH/SLC11A1 |
| 1. **Downregulated genes in peritumoral HSCs vs quiescent HSCs** | | |
| hsa03040 | Spliceosome | BCAS2/CDC5L/SF3A1/SNRNP200/SNRPB2/SNRPE |
| hsa03050 | Proteasome | POMP/PSMA2/PSMC1/PSMD1/PSMD4/SHFM1 |
| 1. **Upregulated genes intratumoral cancer-associated myofibroblasts (CAMFs) vs quiescent HSCs** | | |
| hsa04512 | ECM-receptor interaction | COL1A2/HSPG2/ITGA11/LAMA4/LAMB1/TNC |
| hsa00010 | Glycolysis / Gluconeogenesis | ALDH1A3/ENO1/GALM/LDHA/PFKM |
| hsa04974 | Protein digestion and absorption | ATP1A4/COL12A1/COL6A1/FXYD2/SLC38A2 |
| hsa00100 | Steroid biosynthesis | C5ORF4/DHCR24/DHCR7/EBP/FDFT1 |
| hsa00330 | Arginine and proline metabolism | ADC/ALDH18A1/ALDH1B1/ALDH7A1/ASS1/ |
| hsa04610 | Complement and coagulation cascades | BDKRB1/C1R/C1S/C3/CD46/CFH/F12/PLAT |
| hsa00310 | Lysine degradation | ALDH1B1/ALDH7A1/EHMT2/HADH/PIPOX/PLOD2 |
| hsa04270 | Vascular smooth muscle contraction | ACTA2/ACTG2/ITPR1/KCNMB1/MYL6B/MYL9 |
| hsa01040 | Biosynthesis of unsaturated fatty acids | ACOT7/ELOVL6/FADS2/PTPLA |
| hsa00280 | Valine, leucine and isoleucine degradation | ALDH1B1/ALDH7A1/AOX1/HADH/HIBADH |
| hsa00270 | Cysteine and methionine metabolism | AHCY/LDHA/LDHB/MAT2A/SRM |
| hsa00051 | Fructose and mannose metabolism | ALDOC/PFKM/PFKP/PHPT1//PI1 |
| 1. **Downregulated genes intratumoral CAMFs vs quiescent HSCs** | | |
| hsa04210 | Apoptosis | AKT3/ IL1A/ IL3RA/IRAK2/MAP3K14/TNF |
| hsa04722 | Neurotrophin signaling pathway | AKT3/CDC42/FOXO3/IRAK2/JUN/MAP2K7 |
| 1. **Upregulated genes in peritumoral HSCs vs intratumoral CAMFs** | | |
| hsa04610 | Complement and coagulation cascades | C1QA/C1QB/C2/CFD/SERPINA1 |
| hsa03320 | PPAR signaling pathway | GK/MMP1/PLTP/PPARG/SLC27A1 |
| hsa04062 | Chemokine signaling pathway | ARRB2/CCL14/PRKCB/SHC2/VAV1/WAS |
| hsa04142 | Lysosome | CTSA/CTSD/GBA/MAN2B1/SLC11A1 |
| hsa04662 | B cell receptor signaling pathway | FOS/LILRB3/PRKCB/VAV1 |
| hsa04370 | VEGF signaling pathway | MAPK13/MAPKAPK3/PRKCB/SHC2 |
| hsa04664 | Fc epsilon RI signaling pathway | FCER1G/MAPK13/PRKCB/VAV1 |
| hsa00511 | Other glycan degradation | GBA/MAN2B1 |
| hsa00531 | Glycosaminoglycan degradation | HPSE/SPAM1 |
| hsa04977 | Vitamin digestion and absorption | SLC46A1/TCN2 |
| hsa05200 | Pathways in cancer | DAPK1/FOS/MET/MMP1/PPARG/PRKCB |
| 1. **Downregulated genes in peritumoral HSCs vs intratumoral CAMFs** | | |

| hsa04512 | ECM-receptor interaction | COL1A2/COL6A1/ HSPG2/ITGA1/ITGA11/TNC |
| --- | --- | --- |
| hsa04974 | Protein digestion and absorption | COL12A1/COL4A2/COL5A1/COL5A2/COL6A2 |
| hsa00520 | Amino sugar and nucleotide sugar metabolism | CYB5R2/GFPT1/GNPNAT1/PGM3/UAP1/UGDH |
| hsa00010 | Glycolysis / Gluconeogenesis | ALDH7A1/LDHA/ PDHA1/PFKM/PFKP/PGAM1/ |
| hsa04115 | p53 signaling pathway | CDK6/CHEK1/GADD45A/ IGFBP3/THBS1 |
| hsa00620 | Pyruvate metabolism | ALDH7A1/LDHA/LDHB/MDH2/PDHA1 |
| hsa00052 | Galactose metabolism | B4GALT2/GANC/PFKM/PFKP |
| hsa00240 | Pyrimidine metabolism | CTPS/DTYMK/NME1/NT5E/TYMS/UCK2 |
| hsa04141 | Protein processing in endoplasmic reticulum | BAG2/CRYAB/SEC13/SEC23A/SEC31A/TXNDC5 |
| hsa04610 | Complement and coagulation cascades | BDKRB1/C1R/C1S/CFH/PLAT/SERPINE1 |
| hsa01040 | Biosynthesis of unsaturated fatty acids | ACOT7/FADS2/PTPLA |
