# Supplementary material for: Clinical significance and gene expression study of human hepatic stellate cells in HBV related-hepatocellular carcinoma
Source: J Exp Clin Cancer Res. 2013 Apr 19;32(1):22. doi: 10.1186/1756-9966-32-22 (PMC3654985; doi:10.1186/1756-9966-32-22)
Supplement: Additional file 5: Table S5 — qRT–PCR validated genes in Gene Ontology analysis and pathway analysis in different phenotype cells. [file 1756-9966-32-22-S5.docx]

**Table S5 qRT–PCR validated genes in Gene Ontology analysis and pathway analysis in different phenotype cells**

| **Gene Symbol** | **Genbank Accession** | **Fold Change in Microarray** | **Fold Change in qPCR** |
| --- | --- | --- | --- |
| 1. Validated genes in Gene Ontology analysis in different phenotype HSCs | | | |
|  | | P vs Q | |
| COL1A2 | NM_000089 | 8.56 | 3.45 |
| ACTG2 | NM_001615 | 9.69 | 2.78 |
| TIMP2 | NM_003255 | 3.21 | 2.94 |
| MMP25 | NM_022468 | 2.64 | 4.35 |
| SGK1 | NM_005627 | 3.39 | 4.55 |
| MMD | NM_012329 | 9.27 | 3.85 |
| GPX3 | NM_002084 | 22.16 | 6.67 |
| CCL20 | NM_004591 | -88.78 | 0.11 |
| CD83 | NM_004233 | -3.39 | -2.72 |
| SOCS3 | NM_003955 | -8.71 | -2.43 |
| TLR2 | NM_003264 | -6.65 | -3.64 |
| TNF | NM_000594 | -5.34 | -3.87 |
|  |  | P vs A | |
| IL17RA | NM_014339 | -8.73 | -3.15 |
| CD81 | NM_004356 | -2.56 | -1.69 |
| CXCL2 | NM_002089 | -4.10 | -1.35 |
| F3 | NM_001993 | -44.77 | -4.07 |
| MMP10 | NM_002425 | -3.50 | -1.59 |
| IGFN1 | NM_001164586 | 2.24 | 3.22 |
| 1. Validated genes in Gene Ontology analysis in different activated phenotype cells | | | |
|  | | T vs A | |
| HMGB4 | NM_145205 | 2.57 | 2.30 |
| IL6 | NM_000600 | 190.15 | 5.84 |
| BCL2L2 | NM_004050 | 3.65 | 3.50 |
| TLR5 | NM_003268 | 2.92 | 4.33 |
| CXCL6 | NM_002993 | 31.55 | 3.45 |
| PDGFA | NM_033023 | 3.12 | 2.56 |
| IL17RE | NM_153483 | 3.59 | 2.75 |
| MMP3 | NM_002422 | 38.61 | 2.25 |
| ACTR2 | NM_001005386 | -23.89 | -2.94 |
| AKT3 | ENST00000366539 | -6.64 | -1.77 |
| BMP2K | NM_017593 | -28.87 | -3.34 |
| CCL3 | NM_002983 | -7.53 | -3.89 |
| CD36 | NM_001001547 | -11.09 | -5.50 |
| EGR2 | NM_000399 | -18.41 | -4.76 |
| HMGCR | NM_000859 | -13.45 | -2.82 |
| LPL | NM_000237 | -31.63 | -2.95 |
| MMP10 | NM_002425 | -3.50 | -1.66 |
| VCAM1 | NM_001078 | -44.10 | -7.07 |
| ACTG2 | NM_001615 | 156.00 | 2.41 |
|  | | T vs P | |
| VEGFC | NM_005429 | 23.43 | 2.22 |
| COL5A1 | NM_000093 | 18.30 | 1.86 |
| TGFB1I1 | NM_001042454 | 9.11 | 2.09 |
| ACTA2 | NM_001613 | 37.81 | 1.30 |
| 1. Validated genes in pathway analysis in different cell phenotypes | | | |
|  |  | T vs P | |
| COL1A2 | NM_000089 | 80.21 | 2.66 |
| HSPG2 | NM_005529 | 10.61 | 4.53 |
| ITGA1 | NM_181501 | 26.18 | 2.63 |
| TNC | NM_002160 | 19.92 | 2.5 |
| THBS1 | NM_003246 | 16.87 | 2.72 |
| GK | NM_001128127 | -3.01 | -1.96 |
| MMP1 | NM_002421 | -2.08 | -1.89 |
| PLTP | NM_006227 | -6.28 | -1.89 |
| PPARG | NM_138711 | -3.02 | -2.27 |
| SLC27A1 | NM_198580 | -2.40 | -2.08 |

Abbreviations: HSCs: hepatic stellate cells; A: culture-activated hepatic stellate cells (HSCs); P: peritumoral HSCs; Q: quiescence phenotype HSCs; T: intratumoral myofibroblasts.
